# The gut microbiota and its metabolite butyrate shape metabolism and antiviral immunity along the gut-lung axis in the chicken

**DOI:** 10.1038/s42003-024-06815-0

**Published:** 2024-09-20

**Authors:** Vincent Saint-Martin, Vanaique Guillory, Mélanie Chollot, Isabelle Fleurot, Emmanuel Kut, Ferdinand Roesch, Ignacio Caballero, Emmanuelle Helloin, Emilie Chambellon, Brian Ferguson, Philippe Velge, Florent Kempf, Sascha Trapp, Rodrigo Guabiraba

**Affiliations:** 1 INRAE, ISP, Université de Tours, 37380 Nouzilly, France; 2 Department of Pathology, University of Cambridge, Cambridge, United Kingdom

**Keywords:** Innate immunity, Microbiome, Agriculture

## Abstract

The gut microbiota exerts profound influence on poultry immunity and metabolism through mechanisms that yet need to be elucidated. Here we used conventional and germ-free chickens to explore the influence of the gut microbiota on transcriptomic and metabolic signatures along the gut-lung axis in poultry. Our results demonstrated a differential regulation of certain metabolites and genes associated with innate immunity and metabolism in peripheral tissues of germ-free birds. Furthermore, we evidenced the gut microbiota’s capacity to regulate mucosal immunity in the chicken lung during avian influenza virus infection. Finally, by fine-analysing the antiviral pathways triggered by the short-chain fatty acid (SCFA) butyrate in chicken respiratory epithelial cells, we found that it regulates interferon-stimulated genes (ISGs), notably *OASL*, via the transcription factor Sp1. These findings emphasize the pivotal role of the gut microbiota and its metabolites in shaping homeostasis and immunity in poultry, offering crucial insights into the mechanisms governing the communication between the gut and lungs in birds.

## Abstract (web summary)

Comparison of conventional and germ-free chicken show gut microbiota's significant impact on immunity and metabolism. Key findings include its role in lung mucosal immunity during avian influenza and butyrate's influence on antiviral pathways

## Introduction

The gut microbiota (GM) is a complex ecosystem that coexists in a symbiotic relationship with the host. These microorganisms constituting the GM actively participate in a broad range of physiological functions, including digestion, nutrient absorption, and regulation of the immune system^1,2^. Disruptions in the GM composition have been shown to impact the host’s health in multiple ways, ranging from metabolic disorders to immune system dysfunctions^3^. The GM also modulates the host immune activity, enhancing its function against pathogens while limiting its reactivity to self-antigens^1^. One of the key ways in which the GM modulates the host’s physiology is via secreted molecules such as bacterial metabolites, including short-chain fatty acids (SCFAs). SCFAs are the end-products of bacterial fermentation of dietary fibre, and can play a crucial role in regulating immunity and metabolism in the gut and peripheral tissues^4^. SCFAs can also improve intestinal barrier function, limiting the translocation of pathogenic bacteria and their toxins into the bloodstream^5^.

Recent research suggests that SCFAs have broad, extra-intestinal, and long-lasting biological activity beyond the gut^4,6^. They have specific receptors in other tissues and organs, indicating their relevance to the regulation of whole-body physiology. The periphery, including the liver, adipose tissue, lungs, and brain, is particularly sensitive to SCFAs and other microbial metabolites, leading to the coining of terms such as “gut-lung axis” and “gut-brain axis”^7,8^. Therefore, the regulation of microbiota composition has well-recognised consequences for immune development and overall health in humans and mammalian model species.

Poultry farming is a central pillar of global animal protein production, and poultry health is paramount to the success of this industry. The GM plays a crucial role in chicken health and well-being^9,10^, and its acquisition and development are particularly important in early life^11–13^. After hatching, the microbiota in the chicken’s gut begins to develop, with colonisation by bacteria such as *Lactobacillus* and *Bifidobacterium*^14^. The GM is also seen as essential for the development of the immune system in poultry, and studies have shown that an unregulated or poorly enriched microbiota in early life can retard immune system development and functioning^15^. Moreover, we have previously shown that GM can influence the immune response in the chicken respiratory tract, suggesting the functional existence of a gut-lung axis in the chicken^16^. The chicken respiratory tract is particularly sensitive to viral infections, especially those caused by avian influenza viruses (AIV), a significant cause of morbidity and mortality in commercial and backyard poultry^17^. Understanding the mechanisms through which the GM and SCFAs influence metabolism and immunity in the respiratory tract and other peripheral tissues is an area of active research and would allow the development of probiotics/postbiotics with optimised capacity to enhance immune system functions beyond the gut. Nevertheless, the gut-lung axis in birds is largely unexplored, despite most pathogens in commercial poultry targeting the gut and/or airways.

To define how the chicken GM regulates metabolism and immunity in the gastrointestinal tract and in the periphery, we analysed transcriptomic and metabolic signatures in conventional (CV) and germ-free (GF) chickens. GF birds exhibited significant physiological changes in the caecal compartment, lacking central GM-derived metabolites like SCFAs. SCFA detection in peripheral compartments of CV birds and differential expression of innate immunity genes in GF birds’ peripheral tissues further corroborated the functional existence of a gut-lung axis in birds. In an AIV H7N1 challenge, GF chickens showed different immunoregulatory responses to infection, though clinical outcomes were similar. Finally, butyrate, an SCFA, displayed antiviral effects in chicken respiratory epithelial cells, likely via the regulation of interferon-stimulated genes, particularly *OASL*. The observed antiviral signalling mechanism would involve HDAC inhibition and Sp1-dependent regulation of the *OASL* promoter, shedding light on new mechanisms through which the GM regulates poultry mucosal immunity along the gut-lung axis in the chicken.

## Methods

### Animals

GF chickens were produced following an established protocol^18^. Eggs were obtained from a histocompatible B21/B21 White Leghorn layer chicken line (PA12), sourced from SPF hens at INRAE, Plateforme d’Infectiologie Expérimentale (PFIE, Nouzilly, France). Following surface disinfection, the eggs were incubated in a sterile hatching incubator. Following hatch, GF chicks were transferred to sterile isolators in BSL2 containment. For CV birds, hatched under SPF conditions without specific disinfection, a similar isolator environment was provided from day 1 post-hatching. Both groups received ad libitum access to a commercial diet (Safe 115, Safe-Diets, France). GF bird feed was sterilised by gamma irradiation, and tap water (autoclaved for GF birds) was provided through water dispensers. These conditions were maintained for 21 days. Sterility tests, a combination of molecular methods (broad-range 16S ribosomal RNA gene polymerase chain reaction (PCR)^19^) and microbiological methods (thioglycolate and Brain Heart Infusion (BHI) broths to assess the absence of bacteria and fungi up to 14 days after sampling)^18^, were routinely performed on pooled fecal contents from birds within a given isolator. As for the microbiological analyses, 1 mL of stool was mixed with 9 mL of thioglycolate broth with resazurin, while the remaining sample was mixed with 9 mL of BHI. The tubes were then incubated at 37 °C without shaking for 18–48 hours, favouring the growth of aerobic, facultative aerobic, and non-fastidious anaerobic species, including bacteria, fungi, and yeast. Thioglycolate broth with resazurin, designed for detecting non-fastidious anaerobic bacteria, also facilitated the detection of aerobic bacteria, yeast, and fungi, in compliance with the sterility testing standards of the European, American, and Japanese pharmacopoeias^20–22^. Perturbations in the growth media were visually examined after 18 hours of incubation. After 48 hours, a drop from the BHI fecal-broth media was placed on a glass slide and examined under a microscope (×40 magnification) for the presence or absence of bacteria, yeast, or fungi. If suspicion arose regarding the presence of these microorganisms, a sample from the BHI culture was inoculated onto BHI agar plates and incubated at 37 °C for 18 to 48 hours for further analysis. Swabs from isolators were periodically tested during hatching and post-hatching periods until the end of the experiment, employing the same detection methods for bacteria and fungi described above. All experiments were conducted in compliance with the relevant European and national regulations and authorised by the local ethics committee (Comité d’Ethique en Expérimentation Animale Val de Loire) under the reference number APAFIS#30655-2021042114338110 v1.

### Tissue sampling

At day 21 post-hatching, CV and GF birds (*n* = 8 per group) were sacrificed by cervical dislocation followed by blood withdrawal from the occipital venous plexus. Blood samples were stored in heparin-coated tubes for subsequent plasma preparation. Caecal contents, caeca, spleens, and lungs were immediately collected from each chicken, snap-frozen in liquid nitrogen, and stored at −80 °C until further use for metabolomics analysis and/or RNA and DNA extraction and sequencing.

### Avian influenza virus infection model

The low-pathogenic avian influenza virus (LPAIV) strain A/Turkey/Italy/977/1999 (H7N1)^23^, which causes moderate to severe disease in experimentally infected chickens, including interstitial pneumonia and intestinal infection^24,25^, was used in the AIV challenge infection experiment. Virus stocks were prepared by one round of amplification in 10-day-old embryonated chicken eggs at a multiplicity of infection (M.O.I.) of 2 plaque-forming units (PFU) per egg. Three-week-old immunocompetent/immunoresponsive^26^ GF or CV chickens were housed in BSL2 cabinets under negative pressure with HEPA-filtered air. Briefly, a group of 24 GF and 24 CV birds were equally inoculated intra-tracheally (i.t.) with 0.2 ml of 10^6^ EID5_50_ of H7N1, and 15 GF and 15 CV control (mock) birds were inoculated i.t. with 0.2 ml of PBS. At days 1, 2, and 3 p.i., caecal contents, tissues, and plasma were recovered from a group of animals (*n* = 5–8) for different downstream analyses. Birds were carefully monitored (twice daily) during the course of infection, and diseased birds presenting at least 4 of the following symptoms for 24/48 hours were killed by intravenous pentobarbital injection (humane endpoint): ruffled feathers, tremors, coughing/sneezing, diarrhoea, lethargy or anorexia. Clinical signs were evaluated according to the following score: 0 (no clinical signs), 1 (mild clinical signs), 2 (temporary severe clinical signs), or 3 (dead or euthanized due to persistent severe clinical signs). A scoring system was used to evaluate macroscopic lung lesions as follows: 1 (mild, localised oedema and fibrinous exudate), 2 (moderate oedema and with haemorrhage and fibrinous exudate over ~1/4 of the lung), or 3 (severe haemorrhage and extensive oedema over ~1/2 of the lung).

### Cell culture and in vitro treatment and infection

The spontaneously immortalised chicken respiratory epithelial cell line CLEC213 has previously been obtained from the lungs of histocompatible B21/B21 white leghorn layer chickens (PA12)^27^. Cells were cultured in Dulbecco’s Modified Eagles’s Medium (DMEM) supplemented with 10% fetal calf serum (FCS) (Gibco, UK), 2 mM L-Glutamine (Gibco, UK), and 100 UI/ml penicillin and 100 μg/ml streptomycin (PS, Gibco, UK) at 41 °C in a 5% CO_2_ incubator. For SCFA biological effects, cells were plated at 5 × 10^6^ cells/ml in 12-well plates, incubated with acetate, propionate, or butyrate (sodium salt form, Sigma-Aldrich-UK) at varying concentrations and times. Some experiments included chicken interferon alpha (Yeast-derived, Kingfisher Biotech, USA) and Mithramycin A (Merck Millipore, USA). Cell lysates and RNA were analysed for gene expression and metabolomics. Cell viability and counts were assessed using Spark® Cyto (Tecan, Switzerland) after Trypan Blue (Sigma-Aldrich, UK) addition.

AIV infection experiments using CLEC213 were performed to assess the effects of butyrate pre-treatment for 16 h on viral replication and innate immune gene regulation. To this end, subconfluent CLEC213 monolayers (3 × 10^5^ cells/well in six-wells plates) were pre-treated with butyrate (3 mM) for 16 h and then infected with the AIV strains A/Mallard/Marquenterre/Z237/83 (H1N1)^28^ or A/Turkey/Italy/977/1999 (H7N1)^23^ at a MOI of 0.1 or 0.01 by incubating the cells with a virus dilution prepared in serum-free DMEM for 1 h at 37 °C. Following this 1 h adsorption period, cells were washed with PBS and then overlayed with serum-free DMEM containing TPCK-treated trypsin (0.4 μg/ml final). Viral replication was assessed in supernatants or cell lysates (RNA) from 12 to 48 h post-inoculation *via* titration of supernatants on MDCK cells or by qRT-PCR targeting the M1 coding sequence of gene segment 7^29^. Primers M52C and M253R span conserved sequences in gene segment 7 of influenza A virus and have no homology to nucleotide sequences from other species available from GenBank. Viral load quantification was expressed as PFU/mL or M1 genomic copy numbers. Cellular RNA extractions from infection experiments with the H1N1 and H7N1 strains were also used for gene expression analysis.

### HDAC activity assay

The histone deacetylase (HDAC) inhibitory properties of butyrate are extensively documented in mammals^30,31^. The HDAC-Glo™ I/II assay kit (Promega, UK) was used to measure class I and II HDAC activity in CLEC213 cells according to the manufacture’s protocol. Briefly, the acetylated peptide substrate was added to the cell culture medium in the presence or absence of butyrate, and peptide cleavage requiring deacetylated protease activity was quantified by measuring the release of luminescent aminoluciferin using a GloMax plate reader (Promega, UK). Reduction in relative light units (RLU) correlated with decreased HDAC activity due to the inhibitory properties of the tested molecule. Trichostatin A served as a positive control. Data are presented as RLU or as percentage of HDAC activity, with untreated cells as the reference (100%).

### Metabolomics analysis

Caecal contents, tissues, plasma and cell lysates were specifically prepared for optimised SCFA quantification through proton nuclear magnetic resonance (H1-NMR) according to Hauser et al.^32^. Caecal contents samples (20–50 mg) were homogenised in 1.2 ml of phosphate buffer (0.2 M, pH 7) and a CDCl3/CD3OD (2:1, v/v) solution. After centrifugation (5500 × *g*, 10 min, 4 °C) twice, the aqueous phases were collected, dried using a SpeedVac Vacuum Concentrator, reconstituted in phosphate buffer (0.2 M, pH 7), vortexed, and centrifuged (5500 × g, 15 min, 4 °C). Subsequently, 500 µl of supernatant were transferred into 5 mm NMR tubes, and 100 µl of TSP (1 mM) were added. Tissues (100–200 mg), plasma, and cell lysates were homogenised in 4.85 ml of methanol/water (80/20, v/v) per gram of tissue for 40 s. Next, 2 ml of dichloromethane per gram of tissue were added, and the sample was vortexed (5 s). Finally, 2 ml of dichloromethane and 2 ml of water per gram of tissue were added, the sample was vortexed (10 s), and kept standing at 4 °C for 15 min. After centrifugation (5500 × *g*, 15 min, 4 °C), the aqueous phases were collected. Samples were dried using a SpeedVac Vacuum Concentrator, reconstituted in 200 µl of phosphate buffer (0.2 M, pH 7), vortexed, and centrifuged (5500 × *g*, 15 min, 4 °C). Then, 150 µL of supernatant were transferred into 3 mm NMR tubes, and 50 µL of TSP solution (1 mM) were added to the NMR tube. 1H NMR spectra were obtained at 300 K on a Bruker Avance III HD 600 MHz NMR spectrometer. The “noesypr1d” pulse sequence was used for water signal suppression with a mixing time of 100 ms. A total of 1024 and 256 transients were collected for tissue and caecal samples, respectively, into 64k data points using a spectral width of 12 ppm, a relaxation delay of 15 s, and an acquisition time of 4.5 s. Prior to Fourier transform, an exponential line broadening function of 0.3 Hz was applied to the FID. All NMR spectra were phase- and baseline-corrected and referenced to the chemical shift of TSP (0 ppm) using Topspin (V3.2, Bruker Biospin, Germany). Metabolite concentrations were calculated using the TSP signal at 0 ppm integrating for 9 protons and with known concentrations of different metabolites.

### Gene expression analysis

Total RNA was extracted from cells or tissues using the NucleoSpin RNA II kit (Macherey-Nagel, Germany) with rDNAse treatment to eliminate contaminant DNA. RNA quality and concentration were assessed with a NanoDrop (Thermo Scientific, USA). Reverse transcription of 0.5–1 μg total RNA per reaction was performed using the iScript cDNA synthesis kit (Bio-Rad, USA). Quantitative real-time PCR (qPCR) on a CFX96 machine (Bio-Rad, USA) used iQ SYBR Green Supermix, cDNA, primers (250 nM, Eurogentec, Belgium), and nuclease-free water (Sigma-Aldrich, UK) in a 20 μL reaction volume. CFX Manager software 3.1 (Bio-Rad, USA) analysed the qPCR data. Amplicon size was confirmed by 2% agarose gel. Target gene expression was normalised to chicken glyceraldehyde 3-phosphate dehydrogenase and β-2-microglobulin. Relative normalised expression represented as log_2_ fold change compared to the CV chicken group was calculated using the 2^ΔΔCt^ method. Primer pairs are listed in Supplementary Table 1.

### High-throughput qPCR using the 48.48 Dynamic Array

Quantitative high-throughput qPCR was conducted on the BioMark HD instrument using a 48.48 Dynamic Array (Fluidigm). Total lung RNA (20 ng) was reverse transcribed using iSCRIPT^tm^ Reverse Transcription Supermix (Bio-Rad). Pre-amplification used a 1:10 dilution of cDNA in water with Preamp Master Mix (Fluidigm, #100-5581) in a T100 Thermal Cycler (BioRad), following thermal cycling conditions. Exonuclease I-treated pre-amplified cDNAs were diluted 1:5 in TE. The qPCR employed primers, 2x SsoFast EvaGreen with Low Rox (BioRad 172-5211), and 20× DNA binding Dye (Fluidigm PN 100-7609) on a 48.48 Dynamic Array Integrated Fluidic Circuit (Fluidigm). Thermal cycling conditions were 1 min at 95 °C, followed by 30 cycles. The analysis software 3.1.3 (Fluidigm) was used with a quality threshold, linear baseline correction, and auto (global) quantitation cycle (Cq) threshold method. Data were analysed using the 2^ΔΔCt^ method, expressing results as relative fold change (Fc) compared to a given control. Primer pairs are listed in Supplementary Table 1.

### Knockdown of OASL and SP1 expression in CLEC213 pulmonary epithelial cells using siRNA

To investigate the roles played by *OASL* and *SP1* in the cellular and molecular mechanisms underlying the biological effects of butyrate CLEC213 cells, we opted to knockdown (KD) their expression in these cells using RNA interference methods^33,34^. Optimal siRNA sequences for knocking-down *OASL* (GenBank: NM_001397447.1) and *SP1* (Genbank: NM_204604.2) were determined using a tool available on the website of the siRNA manufacturer (Horizon Discovery, USA). The transfection involved adding 10 pmol of siRNA to the Lipofectamine 2000 (Thermo, USA) and OPTI-MEM 1× (Gibco, USA) mixture, vortexing, and incubating for 15 minutes at room temperature. Then, 50 µL of the mixture was added per well in a 24-wells plate or 200 µL per well for a six-wells plate, one hour before butyrate addition. siRNA efficiency was verified through qPCR by measuring *SP1* and *OASL* expression. siRNA sequences used are listed in Supplementary Table 2.

### RNA sequencing analyses

The total RNA from tissue and CLEC213 cells was extracted using the NucleoSpin RNA Plus, Mini kit for RNA purification with a DNA removal column (Macherey-Nagel, Germany). RNA quality was checked by the Bioanalyzer 2100 system using the RNA Nano 6000 Assay kit (Agilent Technologies, CA, USA). The NEBNext® Ultra™ II RNA Library Prep Kit for Illumina® was used to prepare the library. Raw reads were trimmed with TrimGalore (v0.4.1, parameters: –clip_R2 2)^35^. Trimmed reads were mapped and quantified using STAR (v2.6.1c) and RSEM (v1.3.1) using the function rsem-calculate-expression (parameters: –star –sort-bam-by-coordinate) and the reference file Ensembl annotation release GRCg6a, Ensemble annotation release 98, genome-build-accession NCBI:GCA_000002315.5^36^. Differential gene expression was calculated using DESeq2 (v1.30.0)^37^. The Wald test was used to generate *p* values and log2 fold changes. Genes with an adjusted *p* value < 0.05 and absolute log2 fold change >1 were called as differentially expressed genes (DEGs) for each comparison. GO and KEGG pathways enrichment analysis were done using g:Profiler^38^. An FDR threshold of 0.05 was used for determining GO category overrepresentation. For significant enrichment, Benjamini-corrected value *P* ≤ 0.05 was considered. Gene ontology enrichment analysis was conducted using Ingenuity Pathway Analysis software (IPA, Qiagen, Germany). For such, a confidence-boosting maximum adjusted *p* value threshold was applied: 0.45 for caeca, which included the analyses of the top 244 genes from the caeca RNAseq analyses; 0.4 for lungs, which included the analyses of the top 280 genes from the lungs RNAseq analyses; and 0.1 for spleen, which included the analyses of the top 84 genes from the spleen RNAseq analyses. From these datasets, histograms were generated with GraphPad Prism software (version 6.0, GraphPad software, USA), illustrating the relative expression of a chosen set of genes. The complete list of DEG from RNAseq in CV and GF chickens at 21 days of age are shown in Supplementary Data 1. The complete list of DEG from RNAseq in CLEC213 chicken lung epithelial cells are shown in Supplementary Data 2. Finally, selected DEG from tissue RNAseq used for IPA analysis are listed in Supplementary Data 3.

### DNA extraction and 16S sequencing

Total DNA was extracted from caecal contents using the Dneasy PowerSoil kit (Qiagen, UK) following the manufacturer’s protocol, and 16S (V3–V4 region) amplification was performed as described by Zheng et al.^39^. In the limited cycle 2nd round PCR, sample-specific barcodes were added for multiplexing. Pooled libraries underwent QC before Illumina 2 × 250bp paired-end sequencing for comprehensive V3 and V4 coverage. The FROGS analysis pipeline^40^ was used for clustering paired-end reads with a 0.1 mismatch rate, selecting reads based on an expected size of 300 bp and a total amplicon size of 400–600 bp (mean 460 bp). Swarm^41^ was employed for sequence clustering with aggregation distance parameters of 1 and 1 for denoising and final clustering steps. VSearch removed OTUs, including chimeric sequences (69), and quality control measures were applied, excluding rare OTUs (relative abundance < 0.0005% of total read numbers) and those matching phiX sequences in a specific data bank. OTUs were classified using NCBI BLAST+ search within the Silva SSU 123 database^42^. Diversity assessment included Chao1, Shannon α-diversity, and Bray–Curtis β-diversity indexes. Differential abundances were assessed using the R-package DESeq2^37^, with significant logarithmic fold change ratios determined by Wald tests and Benjamini-Hochberg adjustment for multiple testing (*P* < 0.01).

### Flow cytometry analysis

Spleen samples were obtained from both CV and GF chickens, then homogenised using 40-μm sterile strainers and resuspended in PBS supplemented with 2% FCS and 2 mm EDTA. Next, nucleated red blood cells were partly eliminated using the ammonium chloride-based RBC lysis buffer Hybri-Max™ (Sigma-Aldrich, UK). The resulting leucocytes were carefully layered onto Histopaque 1077 (Sigma-Aldrich, UK) and centrifuged for 30 minutes at 750 g without braking, at room temperature, for enhanced purification of leucocytes and elimination of persistent erythrocytes. The leucocytes were subsequently collected from the cellular interface ring, washed in PBS containing 2% FCS and 2 mm EDTA, and then quantified. Viability of purified leucocytes in each sample was determined using trypan blue dye exclusion with the Spark® Cyto (Tecan, Switzerland) system. Cells underwent staining using a series of chicken-specific monoclonal antibodies alongside their corresponding isotype controls, following established protocols^43^. All antibodies were sourced from Biorad, France. To distinguish monocytes and macrophages in the spleen, we utilised an APC-conjugated antibody (clone Kul01, IgG1) targeting the chicken mannose receptor C-type 1 like B (MRCL1-B) of avian monocytes and macrophages. Additionally, we incorporated staining for MHC-II (clone 21-1A6, IgG1, FITC-conjugated) in this process. T lymphocytes were identified based on the expression of their T-cell receptor (TCR) and coreceptors. For this purpose, we employed monoclonal antibodies against CD4 (clone CT-4, FITC-conjugated), CD8α (clone CT-8, PE-conjugated), or the γδ TCR (clone TCR1, IgG1, APC-conjugated). Chicken thrombocytes were characterised using antibodies recognising the fibrinogen receptor CD41/61 (clone 11C3, IgG1, PE-conjugated). To discern chicken B cells, we utilised an APC-conjugated antibody recognising the antigen Bu-1 (clone AV20, IgG1). Quadrant markers were established based on negative populations and isotype controls. Finally, cell viability was assessed using the fluorescent DNA intercalator 7-aminoactinomycin D (7-AAD, BD Biosciences). Cell acquisition (5 × 10^5^ events) was performed using a BD FACSCanto II cytometer (BD Biosciences), with subsequent analysis conducted using FlowJo 7.5.3 software (TreeStar Inc., Ashland, OR). The percentage of the analysed population relative to total acquired events was employed in graph construction.

### Statistical analysis

Unless otherwise specified above or in the figure legends, data are presented as the median or mean ± SEM. The unpaired Student’s *t* test was employed to compare the means of two independent groups. For three or more independent groups, one-way analysis of variance (ANOVA) was conducted to ascertain statistically significant differences among the means, followed by a Tukey post-hoc test utilising a Studentized range statistic to perform pairwise comparisons between groups. Graph Pad Prism 8.0 software (GraphPad, San Diego, CA, USA) was employed for statistical analysis. Significance levels were set at *p* < 0.05 for most analyses, unless otherwise indicated.

## Results

### The GM regulates a wide array of transcriptional signatures governing innate immunity along the gut-lung axis in the chicken

We started by assessing the caecal microbiota profile of our CV chickens during their first weeks of life using 16S DNA sequencing. Diversity patterns across caecal samples collected at days 7, 14 and 21 post-hatch were summarised using four α‐diversity indices. Notably, a significant increase in α‐diversity from day 7 to day 21 was observed (Supplementary Fig. 1a). On day 21, caecal samples exhibited the highest average α‐diversity values (Supplementary Fig. 1a). Further examination of bacterial family relative abundances between 7 and 14 days highlights the dynamics of taxa appearance and disappearance (Supplementary Fig. 1b). Among these families, *Lachnospiraceae*, whose role in the fermentation of polysaccharides into SCFA is well-documented in mammals^44^, is present at both 7 and 14 days. *Lactobacillaceae*, abundant in very young animals, decreases by 14 days. Few operational taxonomic units (OTUs) show notable enrichment between 14 and 21 days, indicating a slower acquisition of new bacterial taxa. Enriched rare OTUs after 14 days belong to *Ruminococcaceae*, *Lachnospiraceae*, and *Lactobacillaceae* families (Supplementary Fig. 1c), suggesting similarities with bacterial taxa found in other studies in the chicken^13,14,45,46^. β‐diversity measures revealed similar taxonomic profiles within samples collected at each time-point post-hatch, and significant associations were noted between sample type and all four β‐diversity indices (*P* < 0.001; refer to Supplementary Fig. 1d for the distribution of Bray–Curtis distances). Overall, these data highlight that the isolators employed in this study did not impede the development of a complex caecal microbiota in our chicken line.

**Fig. 1:**
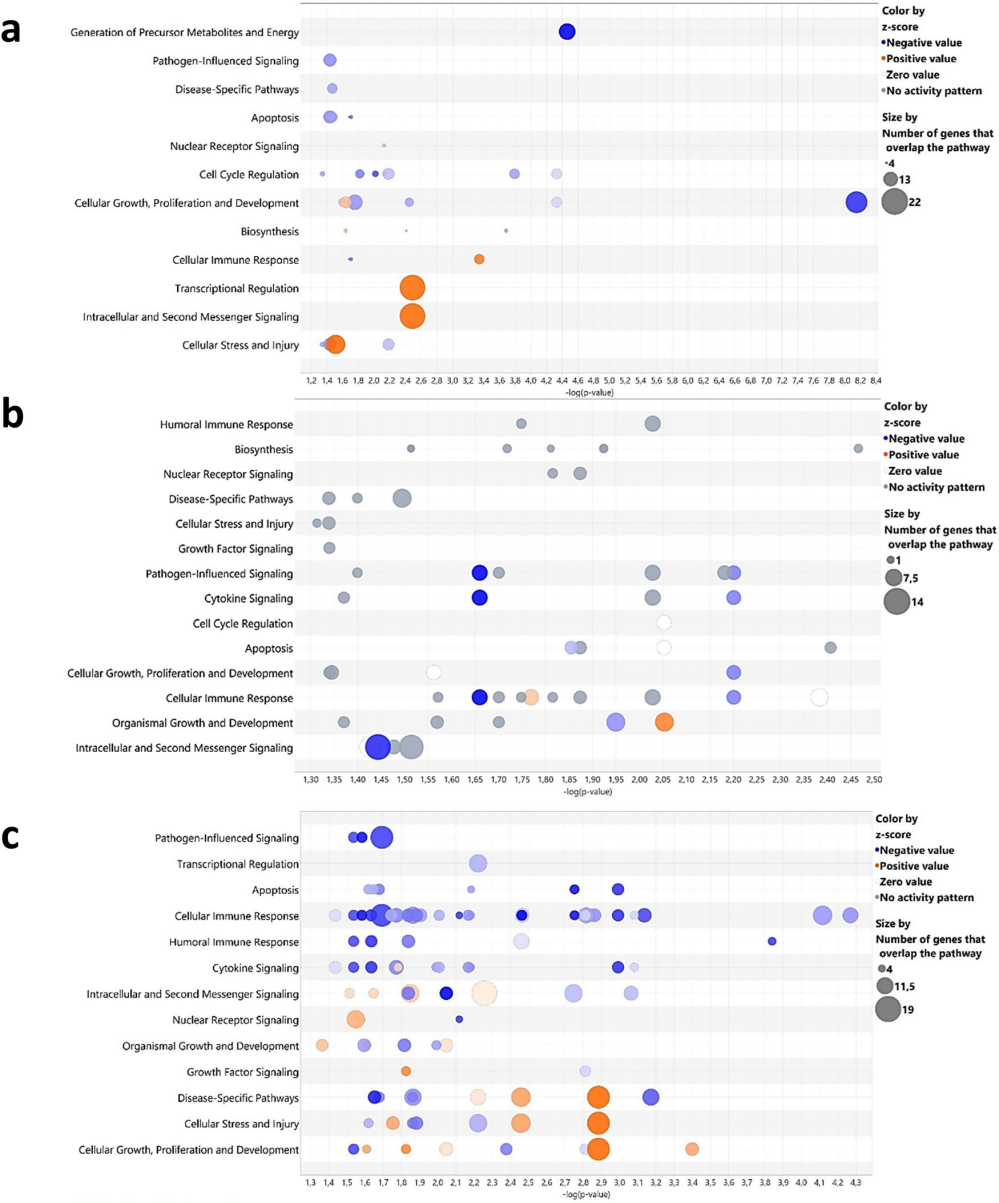
The influence of the gut microbiota on gene expression profiles varies according to the tissue studied. Differently expressed genes (DEG, *p*adj < 0.05, log2 fold change ≥1) from RNAseq data from germ-free (GF) chickens, compared to conventionally raised (CV) chickens at 21 days post-hatch were analysed using Ingenuity Analysis Pathway software, visualised as a “bubble chart.” Circles in each row represent groups of genes associated with a biological function, with size indicating gene count and colour reflecting an intensity gradient ranging from overexpression (orange pattern) to downregulation (blue pattern) in GF chickens using CV chickens as the normalisation controls. The software enables tissue or cell selection and provides inferences on unrepresented genes. A confidence-boosting maximum adjusted *p* value threshold was applied: 0.45 for caeca (**a**), including the analyses of the top 244 genes from the caeca RNAseq analyses; 0.4 for lungs (**b**), including the analyses of the top 280 genes from the lungs RNAseq analyses; and 0.1 for spleen (**c**), including the analyses of the top 84 genes from the spleen RNAseq analyses. Each group comprised caeca, lungs or spleen samples from 5 GF chickens or 5 CV chickens (*n* = 5 biological replicates).

Next, we assessed the impact of GM on chicken immune system development using 3-week-old immunocompetent/immunoresponsive^26^ GF chickens and CV chickens of the same line. RNAseq analysis (*p*adj <0.05, log2-fold change ≥1) revealed significant transcriptional profile changes in GF compared to CV chickens, particularly in the caeca, with ~3000 DEGs showing a downregulation profile, compared to 50 in the spleen and 20 in the lungs. Gene ontology enrichment analysis illustrated disruptions in biological processes, including the cell cycle, biosynthesis, and response pathways to stimuli, emphasising the substantial influence of the GM in the caecal physiology (Fig. 1a). Notable genes that are negatively regulated in GF chickens include *JCHAIN*, a critical factor in mucosal intestinal protection^47^, *NMRAL1*, which regulates innate immunity through NF-κb inhibition and RLR pathways^48^, and *AREG*, which encodes amphiregulin, crucial for maintaining the integrity of the intestinal epithelial barrier^49^. Only a few pathways related to cellular stress or transcriptional regulation showed positive regulation in GF chickens, notably through the sirtuin signalling pathway. Nevertheless, the exact function or expression pattern of these genes or immunological pathways in the chicken are not yet completely resolved, with the exception of *JCHAIN* or *RIGI* (which is absent from the chicken genome)^50^.

In the lung tissue, the variations involved a general downregulation of pathways related to immune responses, development and cell survival in GF chickens (Fig. 1b). Genes affected included those coding for myosin subunits (*MYH1C, MYL3, MYL10*), and those associated with B lymphocyte development or recruitment, such as *CXCL13*, the immunoglobulin Lambda gene, and *FAB4*, implicated in neutrophil recruitment by alveolar macrophages in mouse bacterial pneumonia^51^. However, chickens have rigid parabronchial lungs, harbouring no alveoli, and birds have no neutrophils, possessing heterophils instead, thus making any parallel to mammalian immunological literature a delicate issue^50,52,53^. In the spleen, the main secondary lymphoid organ in chickens, we observed a varying degree of downregulation in immune response processes, including cellular and humoral responses, pathogen-dependent signalling pathways, and cytokine pathways in GF compared to CV chickens (Fig. 1c). Particularly, we observed a significant downregulation of the *MZB1* gene, encoding a protein highly expressed by marginal zone B lymphocytes in the mouse spleen^54^. Additionally, the *KK34* gene, which encodes a cytokine expressed by T lymphocytes in chickens^55^, exhibited downregulation. Nevertheless, the particularities of avian lymphoid organs compared to mammalian species^50^ and how these genes operate within compartments harbouring lymphocytes in the chicken are yet to be described in detail. Furthermore, the antiviral innate response was compromised, with notable downregulation of key genes such as *OASL, IFIT5*, and *IFI6*, all involved in the interferon (IFN) stimulation pathway^56^.

**Fig. 2:**
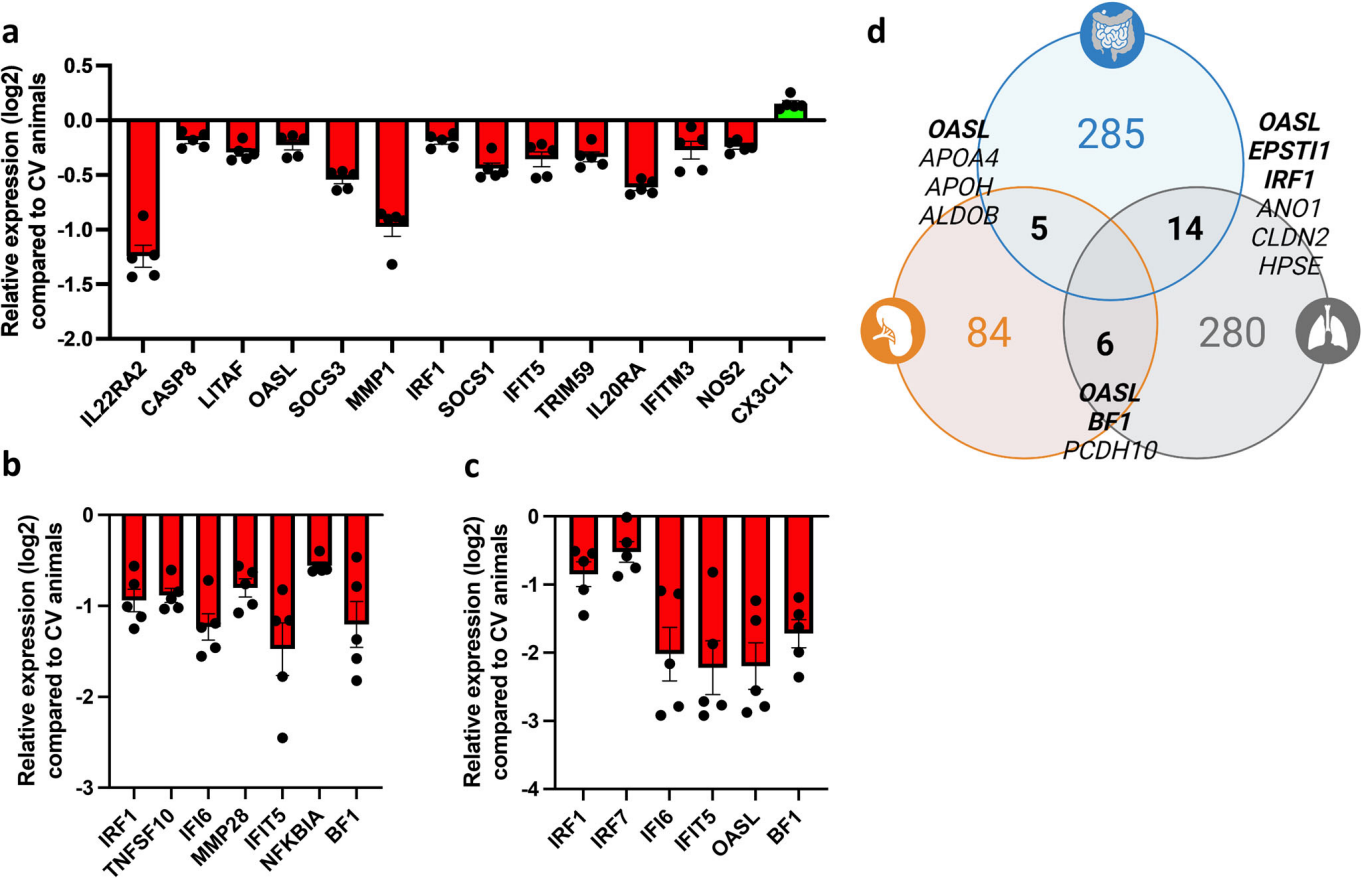
The absence of a gut microbiota results in the regulation of several immune-related genes along the gut-lung axis in the chicken. Differently expressed genes (DEG, *p*adj <0.05, log2 fold change ≥1) associated with immunity and inflammation were identified in the RNAseq analyses of caeca (**a**), lungs (**b**), and spleen (**c**) samples of 21 days-old chickens. This selection included standout genes coding for transcription factors, cytokines, enzymes and other molecules with broad functions in innate and adaptive immunity of vertebrates. Histograms present the relative expression of each gene in log_2_ format for germ-free (GF) animals compared to conventionally raised (CV) animals. A downregulation trend is particularly pronounced in the caeca and moderately in the spleen. In the lungs, contrasting regulatory phenomena with similar intensities coexist. **d** Certain DEGs identified using RNAseq analyses followed by an assessment through the Ingenuity Analysis Pathway software (in which a confidence-boosting maximum adjusted *p* value threshold was applied) are shared by the three organs studied. A Venn diagram illustrates DEG counts per organ, with caeca in blue, lungs in grey, and spleen in orange. Genes shared between organs, where available in the chicken genome, are annotated in the intersections. Immune-related genes, indicated in bold, reveal *OASL* as the sole immune-related gene significantly regulated in all three examined organs. Each group comprised caeca, lungs or spleen samples from 5 GF chickens or 5 CV chickens (*n* = 5 biological replicates). Data are represented as the mean ± SEM.

By closely examining a selection of innate immune-related genes regulated by the GM (*p*adj <0.05, log2 fold change ≥1), we observed a certain overrepresentation of IFN-related genes. In the GF caecal tissue, there was significant downregulation of *OASL, IRF1, IFI6, IFIT5*, and *MX1*, along with genes related to mucosal immune responses (*IL22RA2*, *SOCS1/3*), tissue repair (*MMP1*), and apoptosis (*CASP8*) compared to CV chickens (Fig. 2a). The lung tissue shows a similar trend with significant downregulation of IFN-stimulated genes (ISGs) *IRF1, IFIT5, IFI6*, and *OASL* and NF-κB binding activity regulators (*NFKBIA*), and the canonical MHC class I gene *BF1* compared to CV chickens (Fig. 2b). There was a modest impact on genes within the splenic compartment, with ISGs like *IRF7, IFI6, IFIT5*, and *OASL* significantly downregulated in GF compared to CV chickens (Fig. 2c). Finally, when aggregating DEGs identified through RNAseq analysis, we identified a select group of genes that exhibit consistent downregulation in GF chickens (*p*adj < 0.05, log2 fold change ≥1) (Fig. 2d). Indeed, *OASL* emerges as a primary standout DEG in all three analysed organs.

Attempts were made to complement these data with information on the dynamics of immune-cells populations likely to be regulated by the GM in our model. Specifically, we have tried to perform deconvolution analyses using the R package “granulator”^57^ to access the heterogeneity in the cellular composition of our bulk RNAseq from caeca and lungs, a technique that has been widely used to track compositional alterations of cell types in gene expression data. However, the lack of adequate calibrators for the chicken available in public databases led to our analyses yielding no significant results. We therefore opted to perform a flow cytometry analysis of spleen samples from CV and GF chickens at 7, 14 and 21 days post-hatching. As the main secondary lymphoid organ in chickens, the spleen’s leucocyte provides insightful information regarding the GM’s impact on systemic immunity. Total leucocyte number showed no significant changes between 7 and 21 days-post hatching between CV and GF chickens (Supplementary Fig. 2a). We then applied a staining strategy combining antibodies targeting lymphocytes (CD4, CD8α, γδTCR) and antigen-presenting avian phagocytes (MRCL1-B and MHC-II) (Supplementary Fig. 2b). T CD4 lymphocytes or gamma-delta T cells (CD4+γδTCR+) showed no differences between CV and GF animals independent of the time-point analysed (Supplementary Fig. 2c). The same was observed for CD8α+ cells, which may not only include cytotoxic T cells but also NK cells and dendritic cells^43^. However, MHC-II negative CD8α+ cells, possibly including CTLs and NK cells, presented a significant higher percentage in GF animals at 21 days post-hatching (Supplementary Fig. 2c). Finally, no changes were observed in antigen-presenting avian phagocytes (MRCL1-B+MHC-II+), regardless if they presented high or low levels of expression for MHC-II (MHC-II^hi^ or MHC-II^lo^) (Supplementary Fig. 2d).

Overall, these findings highlight the GM’s significant impact on the transcriptional regulation of IFN-related genes along the gut-lung axis in the chicken, while cellular heterogeneity, at least in the spleen, the main systemic lymphoid organ, remains largely unaffected by the lack of a GM.

### SCFA distribution in the chicken is significantly reduced in peripheral tissues in the absence of a GM

**Fig. 3:**
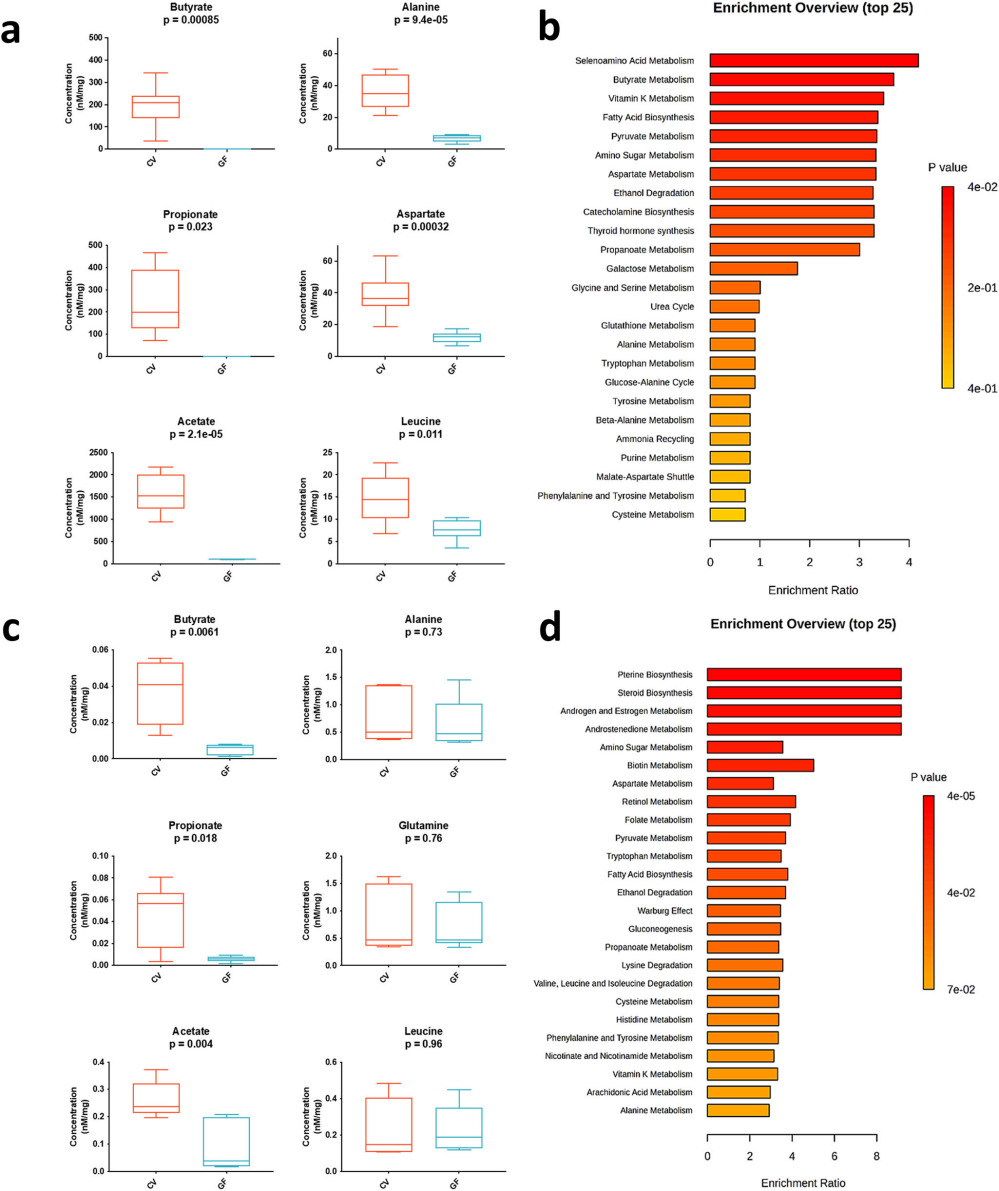
Germ-free chickens show an altered metabolic landscape along the gut-lung axis. We used H1-NMR to analyse metabolite concentrations (nM/mg) in caecal contents (**a**) and lungs (**c**) of 21-day-old conventional (CV) and germ-free (GF) chickens. Metabolic pathway enrichments for caecal contents (**b**) and lungs (**d**) were determined using MetaboAnalyst^103^. This tool identifies essential metabolic pathways (e.g., amino-acid metabolism, biosynthesis, catabolism) across organs. Ratios for each pathway were calculated as “number of metabolites in pathway X”/“Total number of metabolites in pathway X in the database,” and enrichment testing used the “globaltest” method^104^, with resulting *p* values generating colour scales for the histograms. No prior transformations (normalisation, exclusion threshold) were applied to the presented data. Each group comprised caecal contents or lung samples from 6 GF chickens or 6 CV chickens (*n* = 6 biological replicates). Unpaired student’s *t* test was employed for statistical analyses (**a**, **c**). Data are represented as the mean ± SEM.

We have previously established that SCFAs are detectable beyond the gastrointestinal tract in CV chickens and that their concentrations are noticeably reduced or absent in GF animals^16^. Here, our metabolomic analysis corroborates this observation in both caecal contents and lungs (Fig. 3a–d) and extends it to the spleen and serum (Supplementary Fig. 3a–d). More specifically, the concentrations of SCFAs in the caecal contents ranged from 1000–2000 nM/mg for acetate, 100–300 nM/mg for butyrate, and 100–400 nM for propionate. In the lungs, the concentrations found ranged from 0.2–04 nM/mg for acetate, 0.02–0.06 nM/mg for butyrate, and 0.025–0.075 nM/mg for propionate. As expected, the absence of SCFAs-producing microbes in the gut leads to a systemic loss of butyrate, propionate, and acetate across the gut-lung axis, with more pronounced differences in caecal contents (Fig. 3a), although acetate is still present to some extent, probably from residual food sources.

These differences also extend to other metabolites, including amino acids and Krebs cycle molecules (e.g., alanine, aspartate, leucine), abundant in CV animals’ caecal contents, spleen, and lungs but diminished in GF animals (Fig. 3a–d and Supplementary Fig. 3a–d). In caecal contents of GF chickens, pathways related to SCFA metabolism and amino acid biosynthesis are prominently downregulated (Fig. 3b). Serum shows similar profiles, with identified pathways such as vitamin K metabolism and ethanol degradation being altered (Supplementary Fig. 3d). Finally, lung pathways primarily involving amino acid synthesis and essential compounds, such as folate and glutathione, are significantly altered in GF chickens (Fig. 3d), while spleen pathways exclusively encompass alterations in the biosynthesis or metabolism of sugars, nitrogenous bases, and membrane components in the absence of a GM from birth (Supplementary Fig. 3b). The complete dataset of all quantified metabolites is provided in Supplementary Figs. 4 and  5. These observed disparities underscore the GM’s profound influence on chicken metabolism and immune response, providing solid evidence to further explore these regulatory mechanisms in the context of infectious challenges.

### The transcriptional immune response to avian influenza infection in the lung is profoundly regulated by the GM

**Fig. 4:**
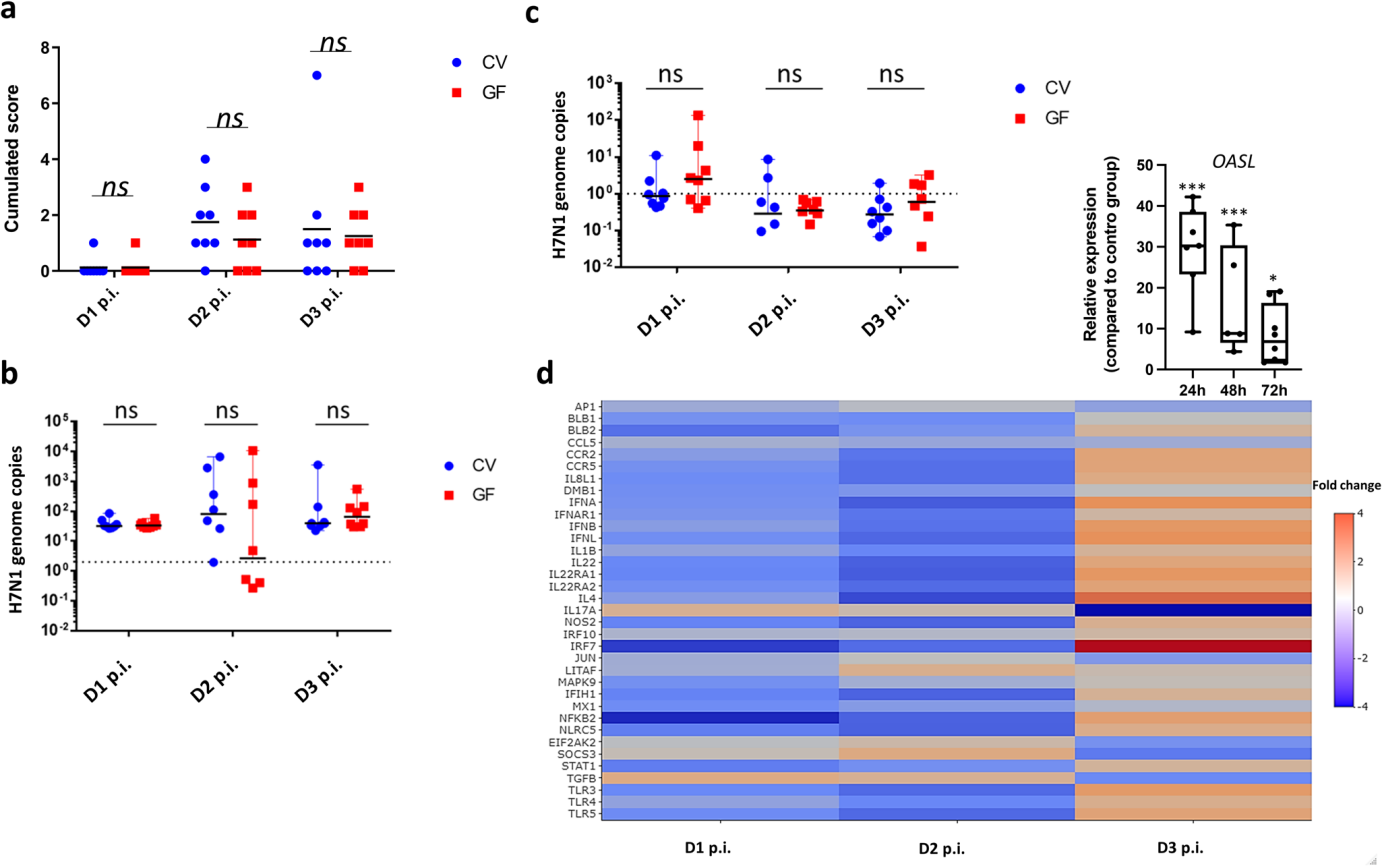
The transcriptional immune response to avian influenza infection in the chicken lung is profoundly regulated by the gut microbiota. Germ-free (GF) animals infected *via* the tracheal route with a 5 × 10^5^ EID_50_ dose of LPAIV H7N1 exhibited a disease profile indistinguishable from their conventional (CV) counterparts. Macroscopic lung lesion scores (haemorrhage, necrosis, oedema) (**a**) and viral genome copies in the lungs (**b**) or in the caeca (**c**) at Days 1–3 post-infection (p.i.) show no significant differences between the two groups. **d** Examination of infection kinetics in GF chickens’ lungs using Medium-Throughput qPCR (Fluidigm) indicates sustained expression of genes associated with inflammation (*IL8L1, IL1B*) and innate antiviral immunity (*IFNA, IFNB, IRF7*, and *OASL* – upper-right box) at Day 3 p.i. The heatmap illustrates relative expression levels in GF-infected animals compared to CV-infected animals on the same day. Each group comprised caeca or lungs samples from 5 mock and 8 infected GF chickens or 5 mock and 8 infected CV chickens (*n* = 5–8 biological replicates), depending on the time-point. **a**, **c**, **d**, each biological replicate is the mean of three technical replicates. Statistical analysis employed unpaired student’s *t* test (**a**–**c**) or one-way ANOVA followed by Tukey multiple comparison test (**d**), where *p < 0.05 and ***p < 0.001. Data are represented as the median (**a**–**c**) or the mean ± SEM (**d**, *OASL* expression).

To define the putative role of the GM in mucosal immunity, we used a H7N1 LPAIV infection model in immunocompetent/immunoresponsive^26^ CV and GF chickens^24,25^. Intratracheal administration of 5 × 10^5^ EID_50_ resulted in nuanced disease with interstitial pneumonia and an innate antiviral response in the lungs. Over a 3-day post-infection period, both CV and GF chickens were monitored closely. Only 3 out of 24 infected animals per group died during the 3-day infection period. There were thus no significant differences between CV or GF animals in terms of mortality. Macroscopic lung lesions were readily observed at 2–3 days post-infection, showing necrosis, haemorrhage, and inflammatory tissue, with low to no mortality observed as mentioned above (Fig. 4a). Despite individual variations, viral replication occurred in the lungs and, to a minor extent, in the caeca (Fig. 4b, c). Surprisingly, we did not observe significant differences in lung pathology or viral burden between CV and GF chickens. Nevertheless, at day 2 post-infection, four out of seven GF birds had undetectable viral RNA loads in the lungs, whereas in the CV group, only one bird showed an absence of detectable viral replication. This suggests that the majority of GF birds may better deal with viral clearance at this specific time-point, although statistical analyses did not reveal significant differences between the two groups (Fig. 4b).

By analysing pulmonary gene expression dynamics during H7N1 infection, we tracked 48 immune-related genes^58^ over a three-day post-infection (p.i.) period in CV and GF animals (Fig. 4d). Disparities emerged in the lungs of GF animals, especially in key antiviral innate immunity genes such as *IRF7, IFNA, IFNB, IFNL*, and *STAT1*, with *OASL* particularly showing a very distinctive regulatory pattern in the absence of a GM (Fig. 4d, upper-right box). These genes showed lower expression at 24 and 48 hours compared to CV animals but a significant elevation at 72 hours, indicating a sustained antiviral response beyond 48 hours in GF animals. These changes also influenced mucosal immunity, as seen in the distinct regulation pattern for *IL22* and its receptors (*IL22RA1, IL22RA2*) in GF animals. Furthermore, reduced expression of anti-inflammatory genes like *TGFB* and *SOCS3* at 72 hours p.i. in GF animals possibly indicates an unresolved inflammatory state. In summary, while the absence of GM does not hinder antiviral responses against AIV in chickens, it influences the quality and amplitude of the immune response at the transcriptional level.

### Butyrate regulates cellular and molecular processes in chicken respiratory epithelial cells

**Fig. 5:**
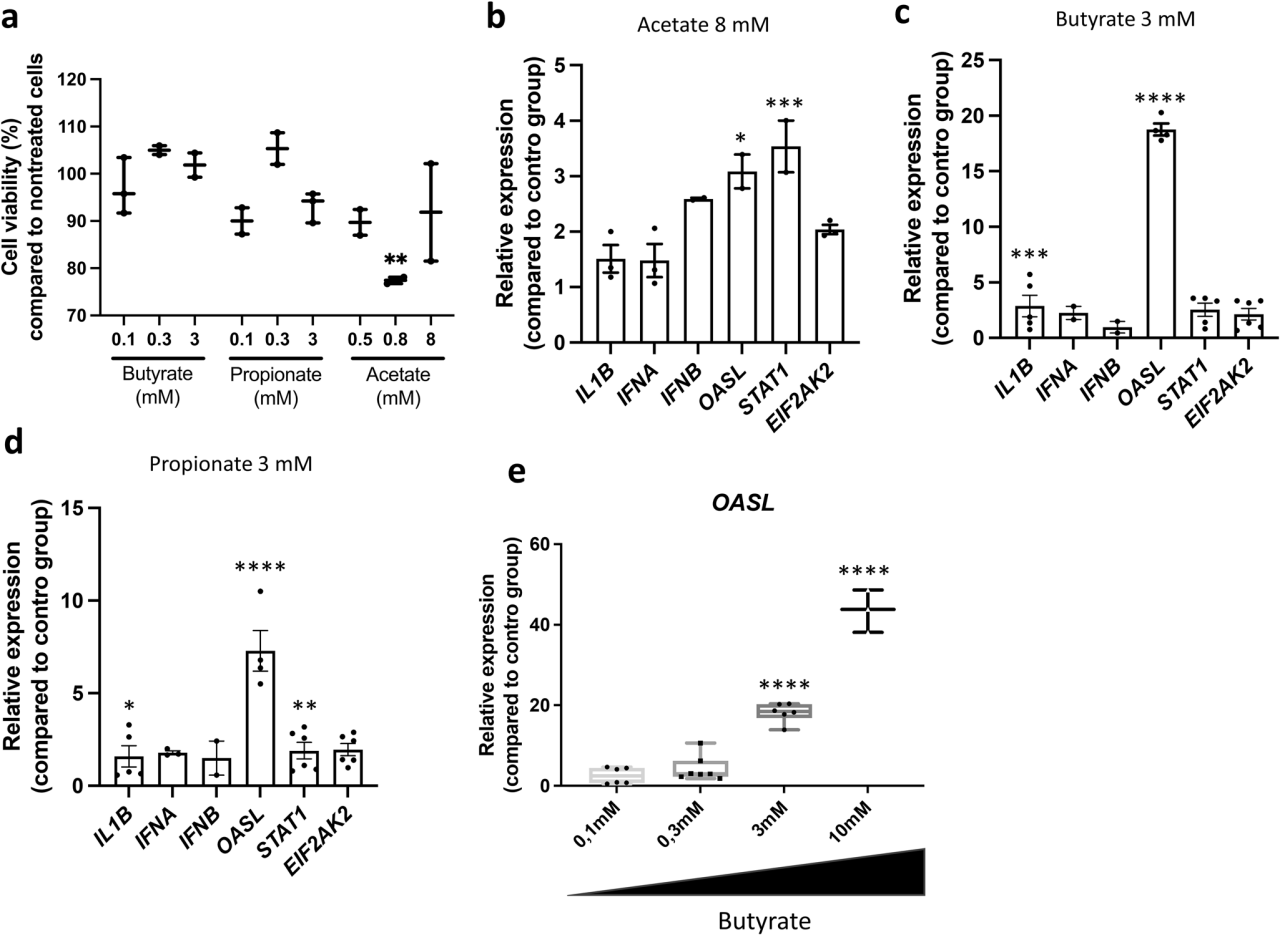
Short-chain fatty acids elicit cellular and molecular responses in chicken lung epithelial cells. The CLEC213 chicken lung epithelial cell line was treated with different concentrations of SCFA for 16 h. Cytotoxicity of butyrate, propionate, and acetate was assessed using a Spark® Cyto and expressed as the percentage of cell viability compared to untreated controls (**a**). qPCR analysis demonstrates that acetate (**b**), butyrate (**c**), and propionate (**d**) elicit different responses in terms of selected innate immune gene expression in CLEC213 cells. Notably, the induction of *OASL* by butyrate exhibits a concentration-dependent relationship (**e**). Statistical analysis was performed using One-way ANOVA followed by Tukey multiple comparison test, with significance levels indicated as follows: *p < 0.05, **p < 0.01, ***p < 0.005, ****p < 0.001. **a**, **b**, an *n* = 3 biological replicates is shown, in which each biological replicate is the mean of three technical replicates. **c**–**e** an *n* = 5–7 biological replicates is shown, in which each biological replicate is the mean of three technical replicates. Data are represented as the mean ± SEM.

Shifting our focus to investigations in our unique chicken respiratory epithelial cell line CLEC213^27^, we aimed to model events in the communication between SCFAs and the respiratory tissue. Cytotoxicity tests on this cell line revealed a wide tolerance to SCFAs (Fig. 5a). Despite concentrations exceeding physiological lung levels in chickens (3–10,000 µM), this concentration range facilitated the examination of SCFA-induced effects on metabolism and transcription. Acetate and propionate alone induced moderate transcriptional responses in CLEC213 (Fig. 5b, d). For instance, the expression of *IFNB, OASL*, *STAT1* (with propionate), and *IL1B* (with acetate) increased by approximately three-fold compared to untreated cells. In contrast, butyrate showed a mean 5-fold increase for the analysed genes (Fig. 5c), with a strong concentration-dependent induction of *OASL* (Fig. 5e).

**Fig. 6:**
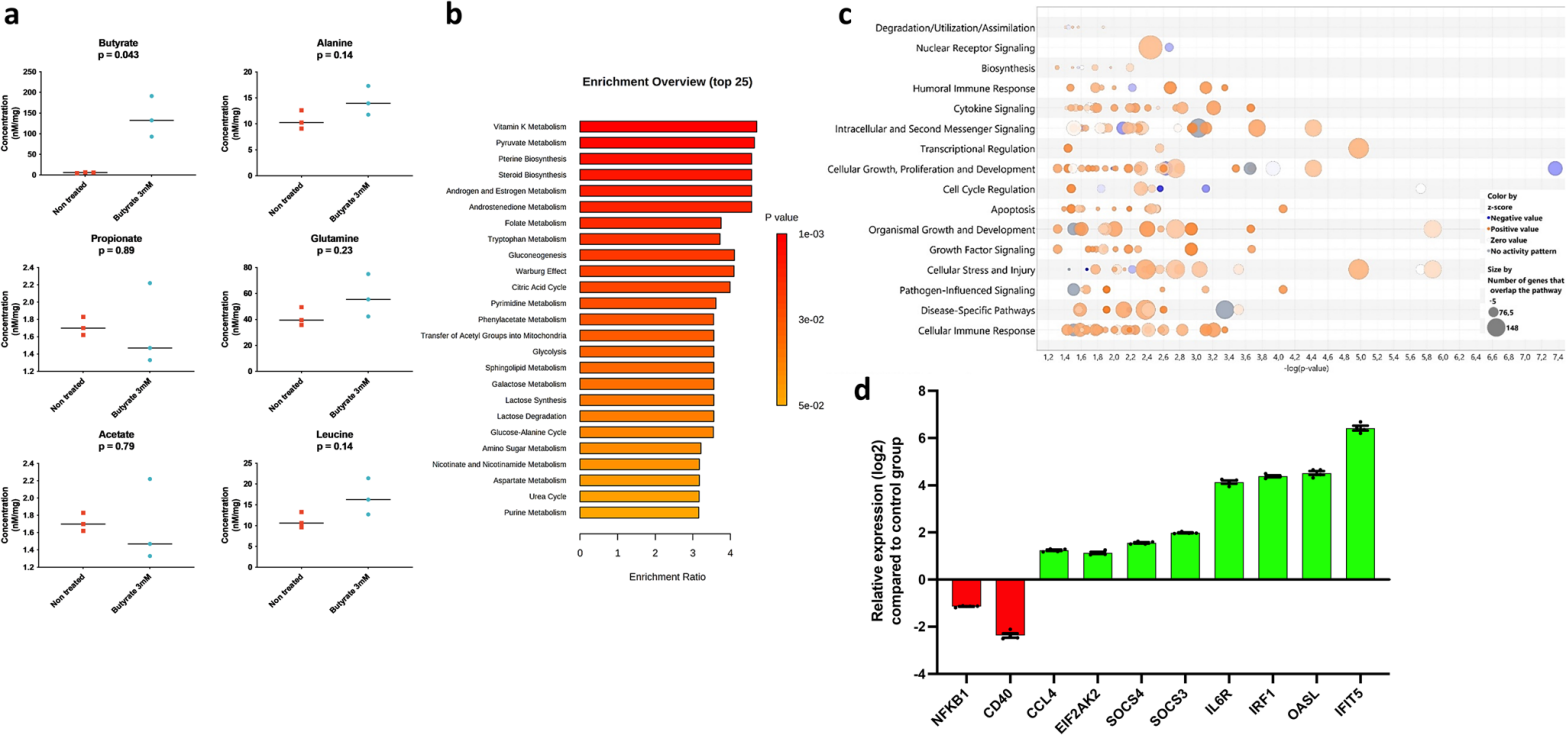
Butyrate regulates metabolic and transcriptional signatures in chicken lung epithelial cells. CLEC213 chicken lung epithelial cells were treated with 3 mM butyrate for 16 h, resulting in an increase in metabolites associated with various biosynthetic pathways (tryptophan, glycogenesis, pyruvate), as analysed by H1-NMR (**a**). Statistical significance was assessed using unpaired student’s *t* test. **b** Metabolic pathway enrichment analysis, employing the “globaltest” method^104^, revealed pathway ratios, with colour scales in histograms corresponding to *p* values. **c** Butyrate induced positive regulation across diverse biological functions in chicken lung epithelial cells, as depicted in a ‘bubble chart’ generated from RNAseq data analysis using Ingenuity Analysis Pathway software. Circles in each row represent groups of genes associated with a biological function, with size indicating gene count and colour reflecting an intensity gradient ranging from overexpression (orange pattern) to downregulation (blue pattern) in butyrate (3 mM) treated cells using untreated cells as the normalisation controls. A confidence-boosting maximum adjusted *p* value threshold of 045 was applied. **d** The log2 relative expression of a group of selected immune-related genes extracted from all DEG from RNAseq data (*p*adj < 0.05, log2 fold change ≥1) in cells receiving butyrate (3 mM) compared to untreated cells at 16 h. **a**, **b** an *n* = 3 biological replicates is shown, while in **c**, **d** an *n* = 6 biological replicates. Data in **a** are represented as the median. Data in **d** are represented as the mean ± SEM.

Next, we further defined the effects of butyrate by analysing the metabolic and transcriptional profiles of CLEC213 cells treated with 3 mM of butyrate for 16 hours. While concentrations of detectable metabolites increased in untreated cells, only butyrate concentrations showed statistically significant differences (Fig. 6a). Enriched metabolic pathways revealed that butyrate functions in CLEC213 cells by enhancing the biosynthesis of essential compounds (e.g., vitamin K, pyruvate, folate, etc.) and amino acids, particularly tryptophan. This suggests a broad impact of butyrate on chicken lung epithelial cells, enhancing their overall metabolic activity (Fig. 6b), consistent with the well-documented effects of butyrate on cellular metabolism^59^.

Ontology enrichment and gene expression analyses revealed that butyrate significantly influences cellular functions in CLEC213 cells, encompassing cytokine signalling, pathogen-influenced pathways, and cellular immunity (Fig. 6c). In addition, a cluster of negatively regulated genes associated with cell growth aligns with butyrate’s role as a histone deacetylase inhibitor (HDACi)^31,60^, inducing cell cycle slowdown or arrest (Fig. 6c). Immune-related gene regulation by butyrate shows a bidirectional pattern, balancing anti-inflammatory and pro-inflammatory signatures. This includes the downregulation of *NFKB1* and *CD40*, alongside overexpressed genes like *SOCS3* and *SOCS4*, known for negative regulation of cytokine signalling (Fig. 6d). Butyrate also exhibits pro-inflammatory actions, as seen in the upregulation of *CCL4*, *IL6R*, and ISGs (*OASL, IFIT5, IRF1, EIF2AK2*), mirroring observations in GF animals (which lack systemic butyrate) and emphasising the role of SCFA in regulating ISGs in the respiratory mucosa.

**Fig. 7:**
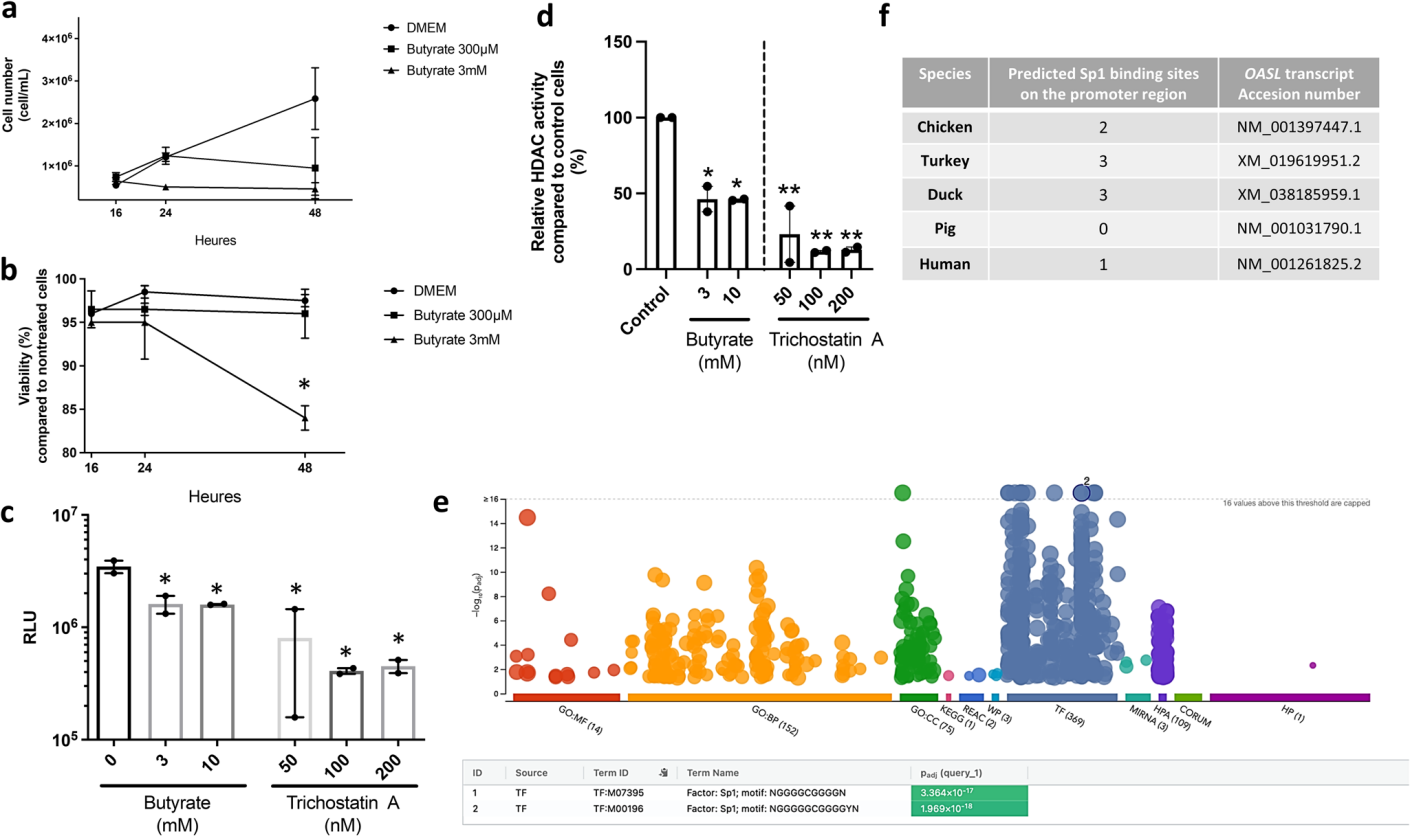
Butyrate possess HDACi functions and strongly regulates *Sp1* in chicken respiratory epithelial cells. Incubation of CLEC213 cells with 0.3 and 3 mM of butyrate for 16 hours resulted in reduced cell counts (per ml) (**a**) with no major impact on cell viability (**b**), as assessed using a Spark® Cyto and expressed as the percentage of cell viability compared to untreated controls. **c** The exposure of CLEC213 cells to 3 mM of butyrate for 16 hours led to diminished histone deacetylase (HDAC) activity as assessed using the HDAC-Glo I/II assay. Data are presented as luminescence ratio (RLU) (**c**) or as the percentage relative to the control group (**d**), with decreased luminescence indicating HDAC inhibition. Trichostatin A (50–200 nM) was used as a positive control. **e** Gene ontology enrichment analysis in butyrate-treated cells (3 mM for 16 h) reveals a significant enrichment (green box) of two Sp1 transcription factor motifs among the top 1000 differentially expressed genes obtained through RNAseq (*p*adj < 0.05, log2 fold change ≥1). Enrichment analysis originally used g:profiler^105^ for molecular function, biological processes, cellular components, various databases, and transcription factors. **f** Identification of potential binding sites for Sp1 in the *OASL* promoter of selected bird and mammalian species. One-way ANOVA followed by Tukey multiple comparison tests (**a**–**d**) was employed for statistical analyses, where *p < 0.05, **p < 0.01. **a**, **b** an *n* = 3 biological replicates are shown. **c**, **d** an *n* = 3 biological replicates is shown, in which each biological replicate is the mean of three technical replicates. Data in **e** are representative of an *n* = 6 biological replicates. Data are represented as the mean ± SEM.

To verify butyrate’s HDACi functions, CLEC213 cells were treated with 0.3 or 3 mM for 16–48 hours. Initial cell proliferation remained unaffected, but between 16 and 24 hours, treatment with 3 mM butyrate inhibited proliferation and led to 15% reduction in cell viability. A similar growth inhibitory profile was also found for 300 µM of butyrate at later timepoints, whilst cell viability remained unchanged at this concentration (Fig. 7a, b). Overall, in the presence of butyrate, the cells reached a steady state where neither the total cell count nor the number of dead cells increased, reflecting a net cessation of cell proliferation (Fig. 7b). Butyrate was shown to inhibit mammalian HDACs from classes I and II^31^. Using the HDAC-Glo I/II assay kit, we measured enzymatic activity in CLEC213 cells. Luminescence counts (Fig. 7c) and percentage of inhibition (Fig. 7d) indicate a significant reduction in HDAC activity following treatment with 3 and 10 mM of butyrate, emphasising its impact on histone deacetylase activity in chickens. Trichostatin A, a known broad-range HDAC inhibitor, exhibits a more pronounced decrease, highlighting butyrate’s HDACi function in CLEC213 cells.

### The transcription factor Sp1 is involved in the regulation of OASL by butyrate in chicken respiratory epithelial cells

Gene ontology analysis from RNAseq data of cLEC213 cells treated with 3 mM butyrate for 16 hours significantly revealed two Sp1 transcription factor motifs (Fig. 7e). In mammals, inhibition of Sp1 with mithramycin A halts TGF-β induction by butyrate in intestinal epithelial cells^61^. We then asked whether this observation could apply to our system. Exploring Sp1-binding sites in the chicken *OASL* promoter, we observed a clear pattern where domesticated bird species have 1–3 Sp1-binding sites, while humans and pigs show none to 1 (Fig. 7f and Supplementary Fig. 6). To test if inhibiting Sp1 mitigates butyrate-induced *OASL* overexpression, we used siRNAs targeting distinct chicken Sp1 transcript regions. siRNA n°2 achieves significant SP1 expression knockdown (Supplementary Fig. 7a), even when cells are treated with butyrate before transfection. In addition, RNAseq analysis of siRNA-transfected CLEC213 cells versus controls highlights *SP1* as the prominently downregulated transcription factor among Sp family members (Supplementary Fig. 7b). *SP1* knockdown significantly downregulated canonical Sp1-regulated genes *LMCD1* and *AQP1*^62,63^, without affecting vital cellular functions in the CLEC213 cell line. To our knowledge, the function of these genes in the chicken are still not resolved, although their conservation among several vertebrate species suggests that they may exhibit overall conserved biological functions.

Transfecting CLEC213 cells with a Sp1-targeting siRNA and treating them with butyrate for 16 hours resulted in a 50% reduction in *OASL* expression, indicating a partial dependence on Sp1 binding to the *OASL* promoter (Supplementary Fig. 7d). This effect did not impact the type I IFN stimulation pathway, as demonstrated by the similar response to IFN-α in transfected and stimulated cells compared to non-transfected cells (Supplementary Fig. 7e). Additionally, mithramycin A, a competitive inhibitor of Sp1 and Sp3, showed cytotoxicity only beyond 0.1 mM, and while it exhibited a non-statistically significant reduction in *OASL* induction by butyrate, it also affected *OASL* induction by IFN-α, suggesting an unexpected interaction between these supposedly independent pathways (Supplementary Fig. 7c).

### Antiviral effects of butyrate in chicken respiratory epithelial cells are partially dependent on the induction of OASL expression

**Fig. 8:**
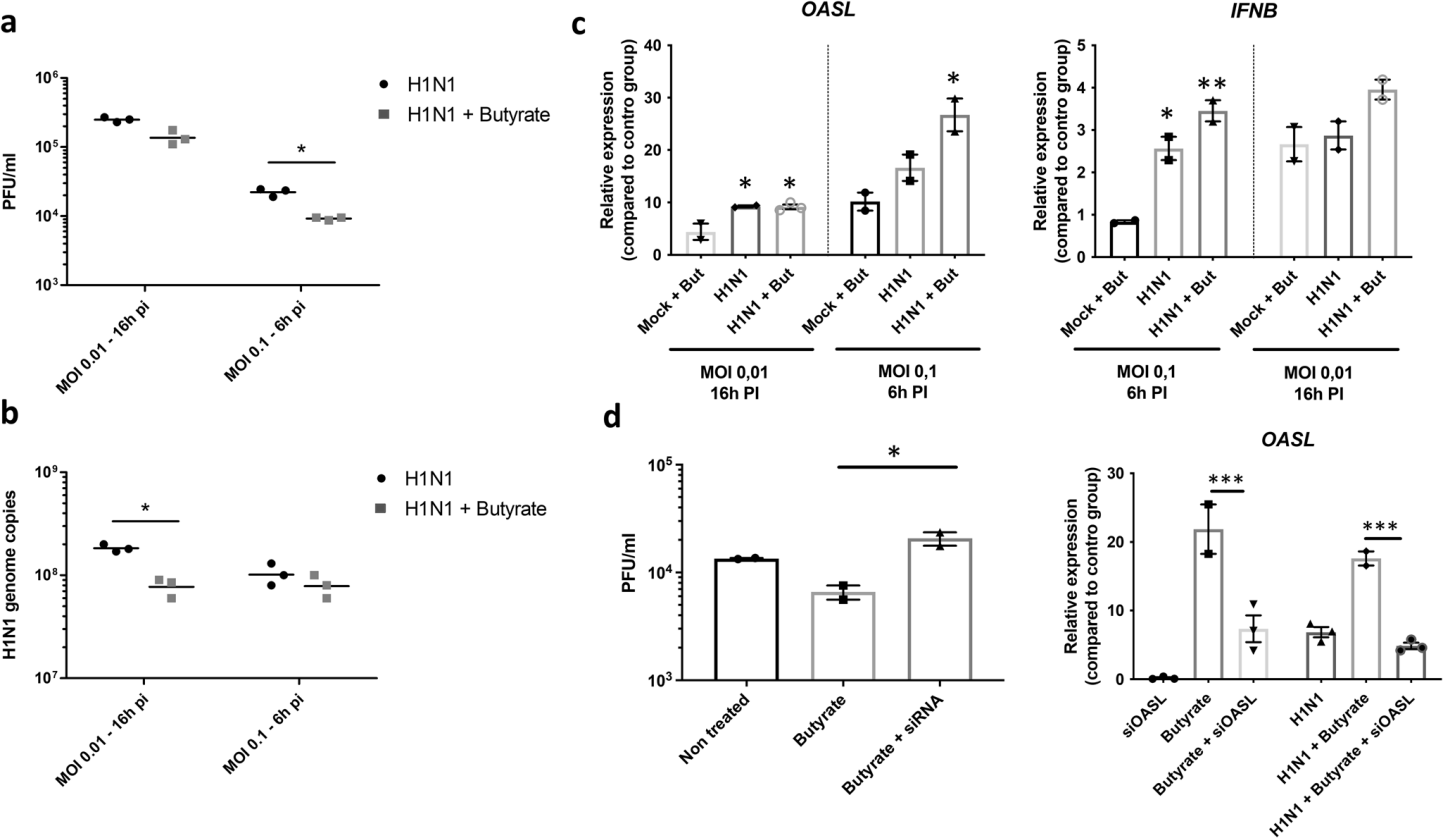
Butyrate enhances viral clearance in chicken lung epithelial cells upon infection with a low-pathogenicity H1N1 avian influenza strain *via* increased type I IFN/ISG responses. Butyrate treatment (3 mM) improves viral clearance in CLEC213 cells infected with an LPAIV H1N1 avian influenza strain. Incubation with butyrate prior to infection (MOI 0.1 for 6 hours or MOI 0.01 for 16 hours) significantly reduces viral titre (PFU/ml) (**a**) and genomic copies (**b**) compared to untreated cells. Additionally, qPCR analysis showed increased expression of *IFNB* and *OASL* in butyrate-treated infected cells (**c**). Silencing *OASL* expression abolishes butyrate’s antiviral effects, resulting in infectious titre identical to untreated cells, concomitantly with reduced *OASL* expression (**d**). Unpaired student’s *t* test (**a**, **b**) and One-way ANOVA followed by Tukey multiple comparison test (**c**, **d**) were employed for statistical analyses, where *p* < 0.05, **p < 0.01, ***p < 0.001. **a** an *n* = 3 biological replicates are shown. **b**–**d** an *n* = 3 biological replicates is shown, in which each biological replicate is the mean of three technical replicates. **a**, **b** are represented as the median. Data in **c**, **d** are represented as the mean ± SEM.

To explore butyrate’s potential antiviral effects in CLEC213 cells, we applied our well-established LPAIV H1N1 (A/Mallard/Marquenterre/Z237/83) infection model^64^, which yields productive infection in CLEC213 cells with mild to moderate cytopathic effects and a low-grade but consistent IFN-I response. Cells pre-treated with 3 mM butyrate for 16 hours, and infected at a MOI of 0.01 or 0.1, exhibited significantly reduced infectious particles, especially at an MOI of 0.1 for 6 h, despite lower infection titre (10^4^−10^5^ PFU/ml) (Fig. 8a). H1N1 genome copies were also significantly decreased (Fig. 8b), while transcription of *OASL* and, to a lesser extent, *IFNB* were significantly enhanced by infection, particularly at an MOI of 0.1 (Fig. 8c). Overall, butyrate pre-treatment led to a reduction in viral load by 50% and amplified *OASL* expression induced by H1N1 infection (MOI 0.1) in chicken respiratory epithelial cells. Interestingly, butyrate was not effective in enhancing *IFNB* expression induced by H1N1 infection (Fig. 8c), which was already low without treatment. We further confirmed the reproducibility of this pattern by repeating the antiviral activity assay with the LPAIV H7N1 (A/Turkey/Italy/977/1999) strain used in the in vivo challenge infection experiment. Infections at an MOI of 0.01 or 0.1 yielded markedly higher viral RNA loads when compared to LPAIV H1N1 infection (Supplementary Fig. 8a). Likewise, butyrate pre-treatment (3 mM) led to a significant reduction in H7N1 genome copies at an MOI of 0.1. Interestingly, H7N1 infection led to enhanced *OASL* and *IFNB* expression levels at both MOIs similar to mock control groups pre-treated with butyrate (Supplementary Fig. 8b, c). Nevertheless, butyrate pre-treatment significantly enhanced both *OASL* and *IFNB* expression in the infected cells to levels higher than those seen in the H1N1 infection experiment at the same conditions (Supplementary Fig. 8b, c), thus confirming the overall capacity of butyrate to limit AIV replication and enhance IFN-I/ISG responses in chicken respiratory epithelial cells.

RNAseq analysis from LPAIV H1N1-infected CLEC213 cells further revealed butyrate’s modulation of infection-related pathways, notably by suppressing HIFs, TGF-β, p53, and NF-κB signalling pathways, emphasising its role as a negative regulator of inflammation during H1N1 infection in chicken lung epithelial cells (Supplementary Fig. 9a). Finally, we developed three siRNAs targeting chicken *OASL* transcripts and used them in CLEC213 cells to target *OASL* expression induced by butyrate (Supplementary Fig. 9b). Subsequent H1N1 infection highlighted *OASL*’s pivotal role in butyrate-induced antiviral effects. qPCR was used to confirm diminished *OASL* expression following siRNA transduction in butyrate-treated cells. In these cells, infectious titres of H1N1 virus were comparable to untreated cells (Supplementary Fig. 9c). Thus, the overall antiviral effects of butyrate would involve enhanced metabolic activity, regulatory influence on inflammatory signals, and pronounced overexpression of ISGs, notably *OASL*.

## Discussion

The establishment of the GM is a critical early event in vertebrate life. Specific bacterial genera and species influence various aspects of poultry physiology^2,12,15,65^. Using a specific pathogen-free (SPF) laying hen model, we aimed to ensure the development of a biologically significant GM before exploring the gut-lung axis mechanisms. Between days 7 and 21, the caecal microbiota saw an enrichment of numerous new species, predominantly within the Firmicutes phylum, including bacteria from the *Lachnospiraceae*, *Lactobacilleae*, and *Ruminococcacae* families. These findings align with Glendinning’s study on broiler chickens (Ross 308) raised without maternal influence from birth and followed until their 35th day of life^66^. Notably, the *Lachnospiraceae* genus *Roseburia*, known for butyrate production, is more prevalent in our animals even at a young age, ensuring significant SCFA production, particularly butyrate. This also emphasises the crucial role of *Roseburia* in poultry mucosal homoeostasis, mainly attributed to the anti-inflammatory properties of butyrate^67–69^.

Comparing metabolites and transcriptional profiles in CV and GF animals highlighted the GM’s profound influence on mucosal homoeostasis in birds. Most of the data available in the literature to discuss these aspects was performed in mammalian model species, especially mice^70–72^. Virtually no mechanistic work has been performed in detail in poultry birds, especially without resorting to antibiotic-induced microbiota suppression^73^. In GF animals, absence of GM leads to altered gene expression and metabolite concentrations in the caecal compartment, signalling changes in intestinal physiology, probably represented by reduced villi, thinner walls, and a more acidic pH as already discussed elsewhere^74,75^. Notably, the near total absence of butyrate and propionate in the caecal contents and in peripheral organs of GF animals confirms their exclusive bacterial origin in our model, since they are unlikely to be produced in significant amounts by the host metabolic machinery^59,76^. Furthermore, the acetate concentrations we observed in GF birds likely originates from dietary sources^77^. Finally, while we consistently observed SCFAs at varying gradients and compartments along the gut-lung axis in chickens as it was observed for mammals^7,78^, we could not elucidate the cellular and molecular mechanisms underlying these systemic distribution. These observations underscore the importance of examining the roles of SCFA in various cell or tissue types along this biological gradient in birds, notably in regard to their epithelial utilisation, integrity, splanchnic utilisation, and peripheral availability, mirroring work performed in mammalian species^78^.

Additionally, the absence of GM results in decreased amino acid concentrations in the periphery, which could directly influence systemic immunity. For instance, the 85% drop in alanine concentration in the caecal contents of GF animals would be crucial for T-lymphocyte activation as suggested elsewhere^79^. Amino acids play pivotal roles in various biological processes essential for immune cell development and function^79,80^. Despite these metabolic disparities, GF animals exhibit no zootechnical differences up to 4 weeks of age^16,18^, suggesting potential compensatory mechanisms for lower nutrient absorption.

The level of knowledge for the molecular mechanisms underlying the communication between the GM and innate immunity in birds does not yet match the level achieved for humans and mice^7,81–83^. Our investigation revealed significant alterations in gene expression signatures due to the absence of a GM in the chicken. In caeca, lungs, and spleen of GF chickens, we observed a substantial reduction in the expression of ISGs, such as *IRF1*, *IFIT*, *OASL*, and *MX1*, aligning with findings in GF mice^84^. Particularly, *OASL*, a pivotal player in chicken antiviral immunity, for example during Newcastle disease virus (NDV) infection^34^, exhibits significant downregulation along the gut-lung axis in our model. In-depth analysis of the chicken *OASL* previously revealed a unique motif, LRLRGG, absent in mammals, which might represent a compensatory mechanism for the lack of ISG15 in birds^85,86^. Analysis of *OASL* sequences from six diverse bird species indicate that these features are likely conserved among avian OASLs. Furthermore, the covalent bonding of avian OASLs to themselves and other immunological proteins, along with structural similarities between avian OASLs’ tandem ubiquitin-like (Ubl) domain and ISG15, suggests a distinctive approach to immune signalling pathways in avian species^85,86^. ISG15 is an important regulator of the mammalian antiviral innate immune response by regulating RIG-I, NF-κB, cytokine and chemokine production, and immune cell activation^87^. However, studies linking the GM and SCFA to the regulation of ISG15 or OASL are currently lacking. Overall, *OASL*’s regulation in GF animals appears markedly altered both at homoeostasis and infection, emphasising its dependence upon positive microbial signals coming from the GM in the chicken. Our comparative study using GF and CV chickens thus provides robust evidence of *OASL* regulation by the microbiota, shedding new light on evolutionary biology and comparative immunology.

Over the past decade, the link between the microbiota and type I IFNs has been extensively documented in humans and mice^88–90^. Recent research has revealed that commensal microbes play a crucial role in maintaining tonic type I IFN levels, which are essential for mounting an effective antiviral immune response. Although specific bacterial strains associated with IFN-I signalling vary among studies, it is noteworthy that the effect of the microbiota is not reliant on a single species. Rather, a diverse community of microbes can reproduce type I IFN responses^84^. These findings collectively support the hypothesis that microbiota-induced type I IFN signalling contributes to the maintenance of mucosal homoeostasis and immune tolerance. Likewise, our findings suggest that at homoeostasis, the innate immune system, including a consistently large panel of IFN-I related genes (e.g., *OASL*, *IFI6*, *IFIT5*), in GF chickens is systematically tuned down due to the physical absence of bacteria in the intestinal tract and the potential lack of microbiota-derived metabolites in the bloodstream. This leads to compromised communication with distant organs such as the lungs, and possibly affects the development of tertiary lymphoid structures in mucosal tissues^15^ and the adequate coordination of antiviral responses.

GF animals infected with an H7N1 AIV strain exhibited clinical manifestations similar to CV animals. Moreover, the virus replicated in the caeca, as described for AIV in domesticated birds^91,92^. Surprisingly, no differences were observed between GF and CV animals with respect to viral replication and clinical outcomes, particularly in regard to lung pathology. This contradicts mouse literature suggesting the GM’s protective role against respiratory viruses^88,93^. Disparities may result from a less effective innate antiviral immune response in the context of GM imbalance, especially in IFN-related genes (*IFNB, IRF7, OASL*, and *MX1*). At 24 and 48 hours p.i., GF animals showed downregulation of most tested genes in the lungs, possibly due to a lack of GM-mediated signals that otherwise prime innate immunity development and triggering threshold. At 72 hours p.i., GF chickens exhibited significant overexpression of ISGs (*OASL* or *IRF7*), potentially aiding in viral clearance. Interestingly, at day 2 post-infection, the lungs of 4 out of 7 GF birds showed nearly undetectable viral loads, while this observation was only made for a single bird in the CV group. This points to a superior viral clearance in the majority of GF birds at this time-point, although statistical analyses did not reveal significant differences between the groups (Fig. 4b). Subsequent analysis of Fluidigm data aimed to explore within-group differences in the relationship between innate immune regulation and viral load, considering the possibility that any lack of significance might stem from a division between responders and non-responders. However, correlations between the two analyses were not identified, possibly due to the limited number of animals used and significant intra-group variability. Despite the observed altered dynamics, both in viral load and gene expression, viral load in GF animals at day 3 post-infection is nearly identical to CV animals. This contrasts with previous findings in antibiotic-treated chickens challenged with a H9N2 LPAIV, where decreased *IFNA* expression and increased viral particle excretion were observed^94^. Regarding *OASL*, its expression decreased by 2 log_2_ at homoeostasis and surged to a level 60 times higher 24 hours post-infection. Among tested genes, only *IRF7* followed a similar kinetics. Therefore, it appears that a delay in triggering the antiviral innate response in the lungs of GF chickens does not impair the proper control of viral load compared to CV chickens. This may be related to the fact that, at homoeostasis, the selected innate immune genes in GF chickens are already at a lower basal level than those in CV counterparts. This could potentially attenuate the exacerbated inflammatory response that leads to severe pneumonia and increased mortality. However, this is not what we observed, as overall lung pathology and clinical scores are similar between CV and GF chickens. Further studies with a broader analysis beyond day 3 are necessary to determine if GF animals can sustain viral load control and if their innate immune response will peak and resolve similarly to CV animals. This remains undefined at present and could lead to the discovery of new molecular mechanisms through which the chicken GM directs innate immunity toward effective and pro-resolutive viral clearance.

A previous report indicated that butyrate influences the response to type I IFN in A549 adenocarcinoma-derived human alveolar basal epithelial cells by modulating the induction of specific ISGs, both positively and negatively, depending on the gene^95^. Unlike the previously documented mechanism of butyrate’s regulation of the type II IFN response^96^, it does not seem to inhibit type I IFN-mediated STAT activation or nuclear translocation. Furthermore, baseline expression of over 30 ISGs was significantly upregulated by at least 4-fold in the presence of butyrate alone, including antiviral restriction factors *OASL* and *BST2*. Consistent with our findings, other researchers also observed that butyrate alters the induction level of several ISGs^95^, revealing a novel mechanism through which butyrate may influence mucosal immunity in the lungs and respiratory virus infections in both mammals and birds. Finally, these results suggest that different subsets of ISGs would have varying requirements for HDAC activity, unveiling a previously unappreciated layer of complexity in the regulation of these genes. The authors, therefore, do not dismiss the possibility that butyrate’s effect on ISGs stems from increased acetylation of non-histone proteins, which are also substrates of HDACs^97^.

Indeed, butyrate’s HDACi properties appears to contribute to *OASL* expression in our CLEC213 cells, albeit showing modest effects in our chicken respiratory epithelial cell model compared to a bona fide HDAC synthetic inhibitor. Unique chicken-specific butyrate functions and the absence of well-characterised GPCR receptors for SCFAs in chickens may contribute to this phenomenon. It was previously reported that more than 20 genes encoding FFAR2/GPR43 paralogs exist in the chicken genome. Although all paralogs seem to encode full-length proteins, the functionality of those genes remains unknown. Moreover, this expansion among FFAR2/GPR43 genes seems to be chicken-specific, as it was not found in other galliform birds such as turkeys and quails^98^. Inspired by studies on butyrate and TGF-β production in mouse intestinal cells^61^, we found Sp1 transcription factor involvement in its mode of action in chicken respiratory epithelial cells. Comparing *OASL* promoter sequences in birds and mammals revealed abundant Sp1 binding sites in birds, a contrast to the scarcity in mammals, emphasising avian immune system peculiarities. siRNA-mediated Sp1 knockdown in CLEC213 cells partially reduced *OASL* expression by 50% upon butyrate treatment, confirming Sp1’s involvement and suggesting a hitherto unidentified mechanism. Sp1 tightly regulates numerous mammalian genes, including both coding and noncoding RNAs, with its expression/activity controlled by various post-translational modifications, such as phosphorylation, acetylation, glycosylation, ubiquitination, and sumoylation^99^. Genetic disruption of Sp1 in mice results in embryonic lethality, highlighting its crucial role in developmental processes^100^.

Finally, butyrate exhibits minor yet noteworthy antiviral effects on chicken respiratory epithelial cells. Butyrate has been shown to directly influence virus replication in certain cases, either independently or in addition to its impact on the type I IFN response, as observed in the case of HIV-1^101^. Moreover, GM-derived butyrate has been recognised for its protective role against influenza infection, enhancing the activity of macrophages and virus-specific CD8+ lymphocytes^81^. Our study corroborates the antiviral activity of butyrate by demonstrating its modest, yet reproducible effect on H1N1 and H7N1 infection in CLEC213 cells, notably through a boost in *OASL* expression. Furthermore, transcriptome sequencing shows butyrate induces wide cellular changes, which possibly contribute to protection against influenza by modifying active infection pathways. Notably, butyrate inhibits the HIF pathway, previously linked to inflammation during H5N1 infection in macaques^102^, potentially mitigating inflammation by preventing HIF activation in pulmonary epithelium. Beyond Sp1, butyrate may regulate *OASL* expression through additional metabolic pathways yet to be defined, highlighting its dual role in shaping immunity and metabolism in our respiratory epithelial system. This unveils a novel mechanism through which a GM-derived SCFA modulates antiviral immunity in peripheral organs of birds. Further investigation into the multifaceted regulation of the type I IFN response by butyrate and its varying effects on virus infection in vertebrates is warranted.

## Conclusions

Our research revealed the intricate interaction between caecal microbiota-derived SCFAs, particularly butyrate, and their role in regulating protective immunity and metabolism across the gut-lung axis in chickens. These insights have broad implications for understanding the gut-lung axis and developing prophylactic strategies in commercial poultry through microbiota modulation. Further exploration of butyrate’s multifaceted effects and its interactions with specific cellular pathways is crucial to harnessing the full potential of SCFAs as next-generation postbiotics for poultry birds.

## Supplementary information

Supplementary Material

Description of additional supplementary files

Supplementary Data 1

Supplementary Data 2

Supplementary Data 3

## Data Availability

The authors declare that all data supporting the findings in this study are available within the article, within Supplementary Data files, or from the corresponding author on reasonable request. All RNAseq data and 16S sequencing data were deposited in the ELIXIR Deposition Database ArrayExpress under the accession numbers E-MTAB-14262, E-MTAB-14264, E-MTAB-14264, E-MTAB-14270, and E-MTAB-14346.
