# Supplementary material for: The gut microbiota and its metabolite butyrate shape metabolism and antiviral immunity along the gut-lung axis in the chicken
Source: Commun Biol. 2024 Sep 20;7:1185. doi: 10.1038/s42003-024-06815-0 (PMC11413219; doi:10.1038/s42003-024-06815-0)
Supplement: Supplementary file 1 — Supplementary Material [file 42003_2024_6815_MOESM1_ESM.pdf]

Supplementary Materials for:

The gut microbiota and its metabolite butyrate shape metabolism and antiviral immunity along the gut-lung axis in the chicken

Vincent Saint-Martin[1], Vanaique Guillory[1], Mélanie Chollot[1], Isabelle Fleurot[1], Emmanuel Kut[1], Ferdinand Roesch[1], Ignacio Caballero[1], Emmanuelle Helloin[1], Emilie Chambellon[1], Brian Ferguson[2], Philippe Velge[1], Florent Kempf[1], Sascha Trapp[1] and Rodrigo Guabiraba[1*]

[1] INRAE, ISP, Université de Tours. 37380, Nouzilly, France.

[2] Department of Pathology, University of Cambridge, United Kingdom.

***Correspondance:** Rodrigo Guabiraba, PhD. Centre INRAE Val de Loire, UMR ISP, 37380 Nouzilly, France. E-mail: rodrigo.guabiraba-brito@inrae.fr

Gene	Forward (5'-3')	Reverse (5'-3')	NCBI Reference Sequence
HPRT	TGGTGGGGATGACCTCTCAA	GGCCGATATCCCACACTTCG	NM_204848.2
IFNA	CAACGACACCATCCTGGACA	GGGCTGCTGAGGATTTTGAA	NM_205427.1
IFNB	TCCTGCAACCATCTTCGTCA	CACGTCTTGTTGTGGGCAAG	NM_001024836.2
IL1B	AGGCTCAACATTGCGCTGTA	CTTGTAGCCCTTGATGCCCA	NM_204524.2
MX1	ACGTCCCAGACCTGACACTA	TTTAGTGAGGACCCCAAGCG	NM_204609.2
OASL	CTTCGGAGTCAGCATCACCA	TCCTGAATCACCTGCCCCAG	NM_001397447.1
EIF2AK2	GGGACATGATTGAGCCAAAGCAAGA	GAGCGTGGGGGTCTCCGGTA	NM_204487.3
M1	TCTAACCGAGGTCGAAACGTA	CAAAGCGTCTACGCTGCAGTCC	
B2M	CGTCCTCAACTGCTTCGCG	TCTCGTGCTCCACCTTGC	NM_001001750.4
GAPDH	GTCCTCTCTGGCAAAGTCCAAG	CCACAACATACTCAGCACCTGC	NM_204305.2
CCL4	CCTGCTGCACCACTTACATAACA	GCGCTCCTTCTTTGTGATGAA	NM_204720.3
CCL5	TATTTCTACACCAGCAGCAAATG	GCAGACACCTCAGGTCC	NM_001045832.2
CCR2	ATGCCAACAACAACGTTTGA	TGTTGCCTATGAAGCCAAA	NM_001045835
CCR5	GTGGTCAACTGCAAAAAGCA	GCCCGTTCAACTGTGTCG	NM_001271141.1
IL8L1	CAAGCACGTTCAGCGATT	ATTCTTGCAGTGAGGTCCG	NM_205018.2
IL12B	CCTGCCTGTCTGCTAAGACC	ATCATTTGCCCATTGGAGTC	NM_213571.2
SOCS3	CTTCGACTGCGTGCTGAA	AGTCTTACGGCAGAGGTGCT	NM_204600.2
IL17A	ATGGGAAGGTGATACGGC	GATGGGCACGGAGTTGA	NM_204460.2
IL18	GCTTGTGGTTCGTCCAGATT	CATTCCACTGCCAGATTTCA	NM_204608.3
IL1B	AGGCTCAACATTGCGCTGTA	CTTGTAGCCCTTGATGCCCA	NM_204524.2
IL4	AACATGCGTCAGCTCCTGAAT	TCTGCTAGGAACTTCTCCATTGAA	NM_001398460.1
IL22	TGTTGTTGCTGTTTCCCTCTTC	CACCCCTGTCCCTTTTGGA	NM_001199614.1
IL22RA1	CTCAGACCTCCGAGCAAAGC	GTGGTCTATGCCATCGACACA	NM_001389540.2
IL22RA2	TGCGACCTGACAGAGGAGACT	AGCTTGCACCCTGCCATAGT	XM_046915187.1
TGFB1	CGACCTCGACACCGACTACT	CCACTTCCACTGCAGATCCT	NM_001318456.1
IFNAR1	GATCTGGCACCCTCGACTTT	TGTTTACCGCCAGCTGTTCC	NM_204859.2
IRF7	TGCCTCAGGCGTCCCCAATG	TGTGTGCCCACAGGGTTGGC	NM_205372.2
MX1	GGACAAGGATGAGGCAGCAG	GCAGGCAACATCAGGTCGTT	NM_204609.2
IFNL3A	TGAGCTGGACCTCACCATCA	GGGCTGTTGGCACGTCTCT	NM_001128496.1
IFIT5	ATGAGTACCATTTCCAAGAATTCCTT	CAATCTGATCCTCTATTGATTCTTCCA	NM_001320422.2
IRF9	GGGATGCAGAGAAGGATGAGAA	GCTCTCCTCGGCCACATG	NM_204558.2
LITAF	CACCAGGCTTCCCTTCTGAG	GGCAGGAAAGCCACTAGGAG	NM_204267.2
BLB1	GTGAGCCGCAAGCTGAATAC	ACCGTGAAGGACTCCACAAC	NM_001044679.2
BLB2	ATGAATGAAGTGGACAGGGTCT	TTCAGGAACCACTTCACCTCG	NM_001318995.3
DMB1	CGAGGTGAAGTGGTTCCTGA	CAGTCCCCGTTCTGCATCA	NM_001312902.2
NOS2	CCACCAGGAGATGTTGAACTATGTC	CCAGATGTGTGTTTTCCATGCA	NM_204961.2
JUN	CGAGCCCCCGGTGTATG	TTGTAGTTGGGTGCAGAGTTGAG	NM_001031289.2
MAPK9	TTACAGAGCGCCAGAGGTTA	TCTCCCATGATGCAACCAAC	NM_205095.2
IFIH1	CGCGACCCCGGATGGTTCAC	GTCTCAATCCCAACCAGGTCCTCC	NM_001193638.2
MMR1L4	GGAACTGCAGGCAGCATATGT	CACAAGGTGCACCAAAATTATTTC	NM_001319013.2
NFKB2	TGAGGTGCGGTTCTATGAGGAT	GGACGGTCAATTTTGGGCTT	NM_204413.2
NLRC5	AGAGCCCTGGGTATGTAAGTTGAG	TTGCATATCATGATCGCAGTGA	NM_001318435.2
TLR3	AACACCCCGCCTAAATATCA	CCACCCTTCAAAATGGATGA	NM_001011691.4
TLR4	ATCTTTCAAGGTGCCACATC	GGATATGCTTGTTTCCACCA	NM_001030693.2
TLR5	TGCACATGTTTTCTCCTAGGT	CCACATCTGACTTCTGCCTTT	NM_001398059.1
AP1	TCCCCTGTCCCCTATTGACA	CGCCGCAATTCTGTTTCTCA	NM_001031289.2

Supplementary Table 1. List of primers used for conventional qPCR (in orange) and Fluidigm (in green) analyses. Primers targeting the Influenza A virus M1 gene were only used in conventional qPCR (in black).

**Target**	**5'-3' sequence**	**3'-5' sequence**
OASL n°1 NM_001397447.1	GGACAGUAACAAGACCACATT	UGUGGUCUUGUUACUGUCCTT
OASL n°2 NM_001397447.1	GUAUUUACUGGGAGAAGUATT	UACUUCUCCCAGUAAAUACAG
OASL n°3 NM_001397447.1	CUGUGAAGGUGCAAGUGAATT	UUCACUUGCACCUUCACAGGT
SP1 n°1 NM_204604.2	GCGGGAAGGUGUACGGCAAUU	UUGCCGUACACCUUCCCGCUU
SP1 n°2 NM_204604.2	GGAGAUGGCAGCAGUGACAUU	UGUCACUGCUGCCAUCUCCUU
SP1 n°3 NM_204604.2	GCACGAACCAGCAGAUCAUUU	AUGAUCUGCUGGUUCGUGCUU

Supplementary Table 2: Sequences of siRNA targeting chicken *OASL* and *SP1*.

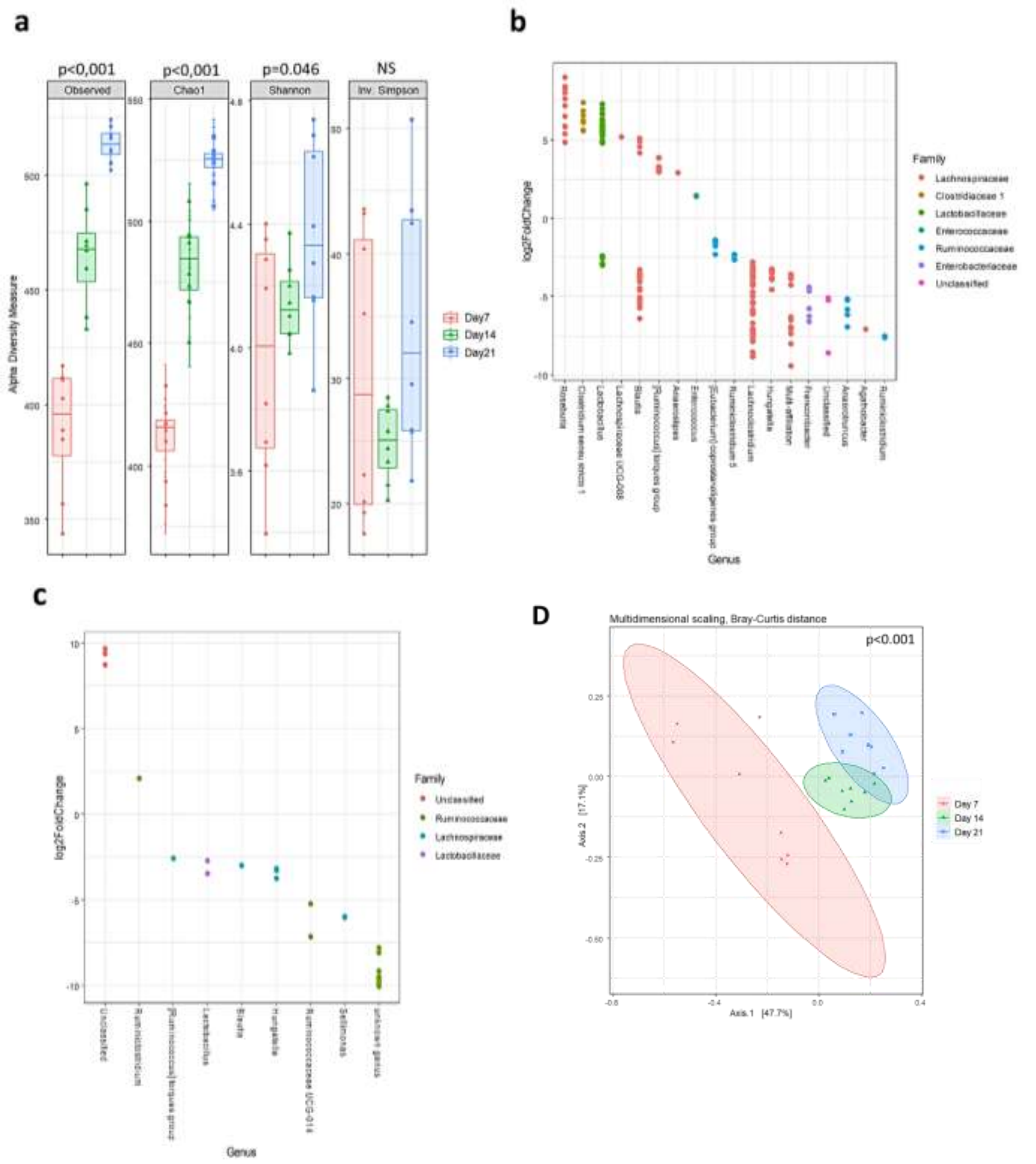

Supplementary Figure 1: Gut microbiota richness and diversity in PA12 laying hens experiences a substantial surge from 7 to 14 days post-hatch, followed by a gradual deceleration between 14 and 21 days. The caecal microbiota profile in conventional (CV) chickens was assessed during first weeks post-hatching using 16S DNA sequencing. (**a**) Various alpha diversity indices, such as Chao1 and Shannon, are computed to assess sample richness, incorporating relative species abundance and a probabilistic approach. All tests, except Simpson, clearly reveal richness disparities among the three age groups. (**b**) Operational Taxonomic Unit (OTU) abundance differences at 7 and 14 days are depicted in columns representing genetically related bacterial species. Positive values in panel b indicate family enrichment at Day 7, while negative values suggest enrichment at Day 14. (**c**) Positive values show Day 14 enrichment, and negative values show Day 21 enrichment. (**d**) Multidimensional scaling summarizes Bray–Curtis distances among samples (beta diversity). Each group comprises 6-8 animals per group (n=6-8 biological replicates), depending on the timepoint evaluated.

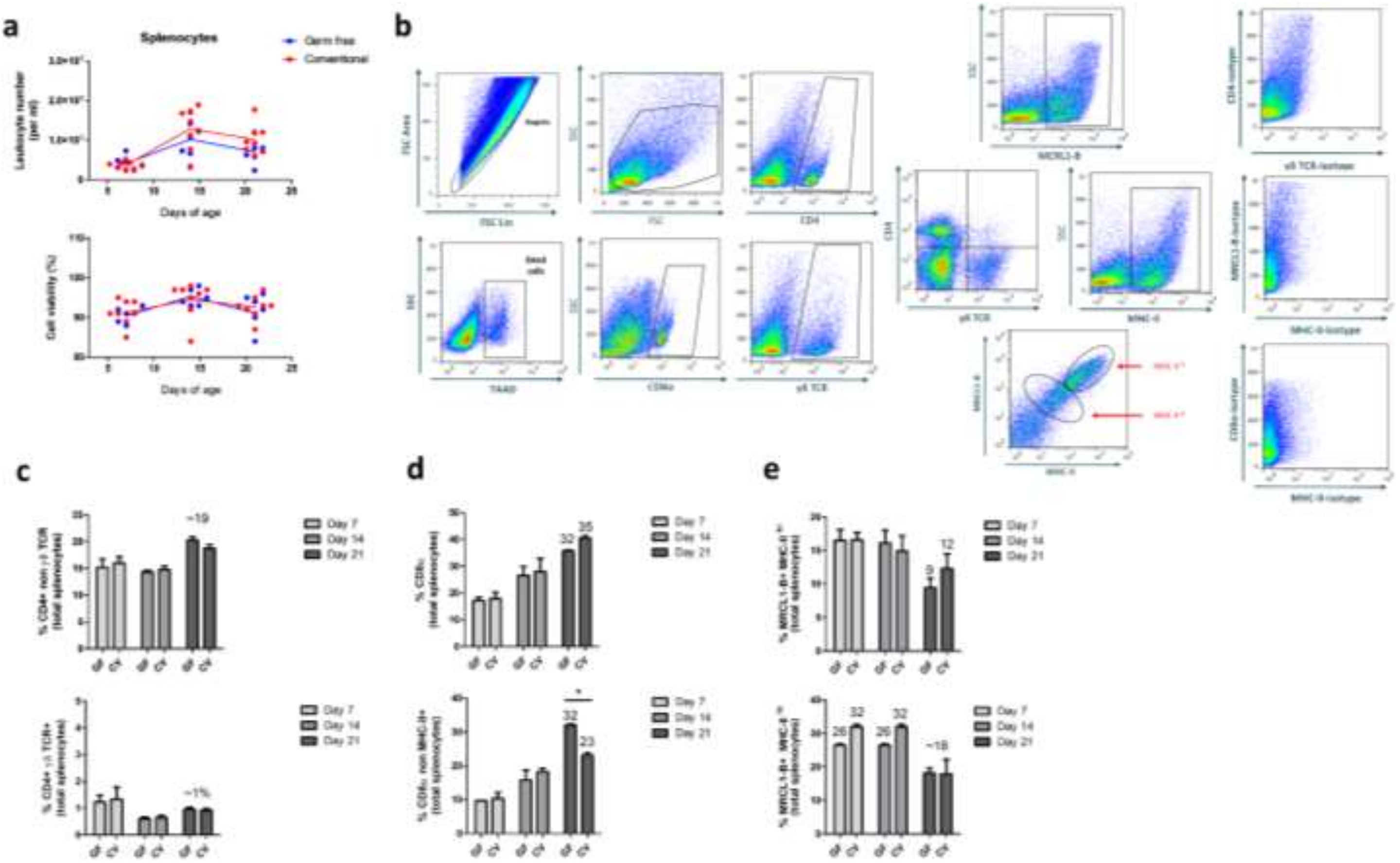

Supplementary Figure 2: Flow cytometry analysis of cells purified from spleen samples of conventional and germ-free chickens at different ages. Spleens were collected from conventional (CV) and germ-free (GF) chickens at 7, 14 and 21 days post-hatching. Cells were freshly purified using Histopaque-based density gradient centrifugation. (**a**) Total leukocyte number (per ml) and cell viability (%) between CV and GF chickens. (**b**) Flow cytometry gating strategy used for the analysis of splenocytes that underwent antibody staining for the identification of avian lymphocytes (CD4+, CD8α+, γδTCR+, CD4+γδTCR+) and antigen-presenting avian phagocytes (MRCL1-B+, MHC-II+, MRCL1-B+MHC-II+hi, MRCL1-B+MHC-II+lo). FSC-Area/FSC-Linear doublet discrimination (singlets) ensured that mostly single cells were selected for analysis. This was flowed by exclusion of dead cells using the fluorescent intercalator 7AAD, that undergoes a spectral shift upon association with DNA. Finally, forward versus side scatter (FSC vs SSC) gating is commonly used to identify cells of interest based on size and granularity (complexity). Isotype controls are also represented in the overall strategy. (**c**) Percentages of total non-γδTCR+ CD4+ (helper T lymphocytes) and γδ T cells (CD4+γδTCR+) between CV and GF chickens. (**d**) Percentages of total CD8α+splenocytes (mainly including cytotoxic T cells, but also NK cells and dendritic cells) and non-MHC-II+ CD8α+splenocytes (potentially excluding dendritic cells). (**e**) Percentages of total antigen-presenting avian phagocytes that are MRCL1-B+ and presenting high (MHC-II+ hi) or low (MHC-II+lo) levels of MHC-II expression. Occasional numbers on the top of histograms are mean percentage values. In (**a**), data are presented as the median (n=6-8 biological replicates). In **c-e**, data are presented as the mean ± SEM (n=6-8 biological replicates). Data were analysed using Unpaired student's t-test (**c-e**) and One-way ANOVA followed by Tukey multiple comparison test (**a**), where: * $p < 0.05$.

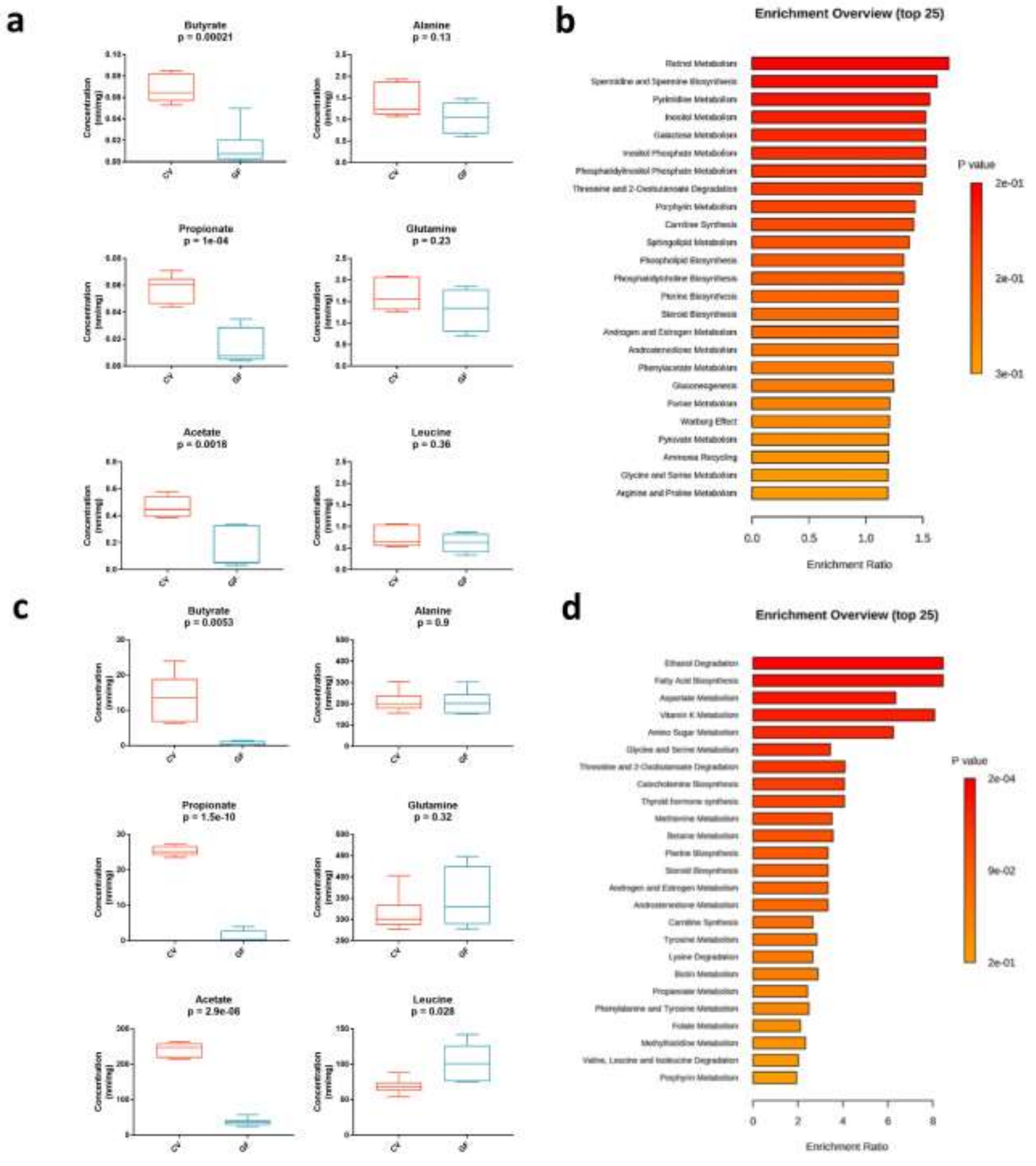

Supplementary Figure 3: Germ-free chickens show an altered metabolic landscape along the gut-gut lung axis. We used H^1-NMR to analyse metabolite concentrations (nM/mg) in **spleen** (**a**) and **serum** (**c**) of 21-day-old conventional (CV) and germ-free (GF) chickens. Metabolic pathway enrichments for **spleen** (**b**) and **serum** (**d**) were determined using MetaboAnalyst [1]. This tool identifies essential metabolic pathways (e.g., amino acid metabolism, biosynthesis, catabolism) across organs. Ratios for each pathway were calculated as "number of metabolites in pathway X" / "Total number of metabolites in pathway X in the database," and enrichment testing used the "globaltest" method [2], with resulting p-values generating colour scales for the histograms. No prior transformations (normalization, exclusion threshold) were applied to the presented data. Each group comprised spleen or serum samples from 6 CV chickens and 6 GF chickens (n=6 biological replicates). Unpaired student's t-test was employed for statistical analyses (**a**, **c**). In panels **a** and **c**, data are represented as the mean ± SEM

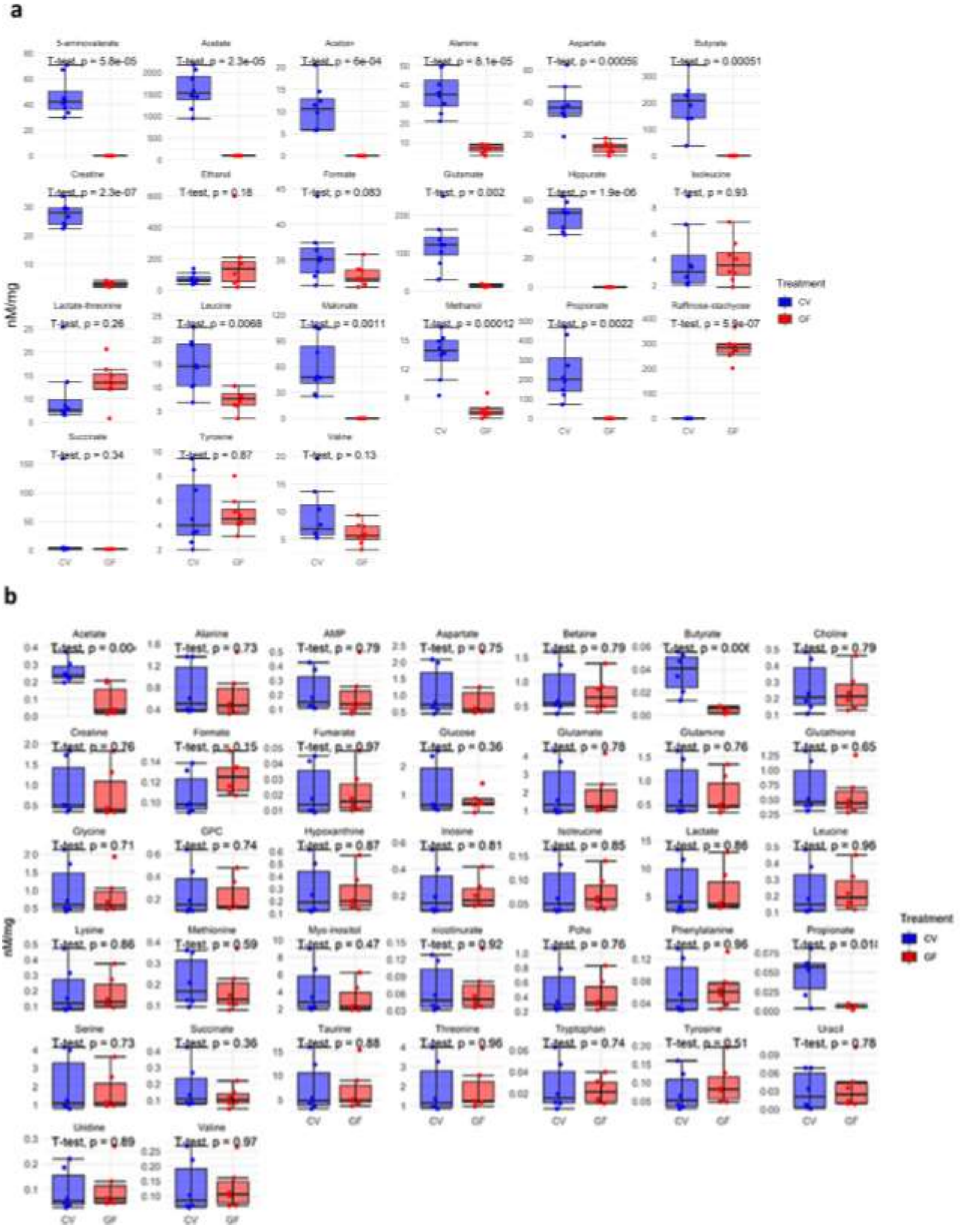

Supplementary Figure 4: Complete H[1]-NMR dataset for the quantification of metabolites in chicken mucosal tissues. We used H[1]-NMR to analyse metabolites concentrations (nM/mg) in **caecal contents** (**a**) and **lungs** (**b**) of 21-day-old conventional (CV) and germ-free (GF) chickens. Statistical comparisons of means for each metabolite were performed using an unpaired student's t-test, and p-values are indicated below the respective metabolite names. Each group comprised caecal contents or lung samples from 6 CV chickens and 6 GF chickens (n=6 biological replicates). Data are represented as the mean ± SEM.

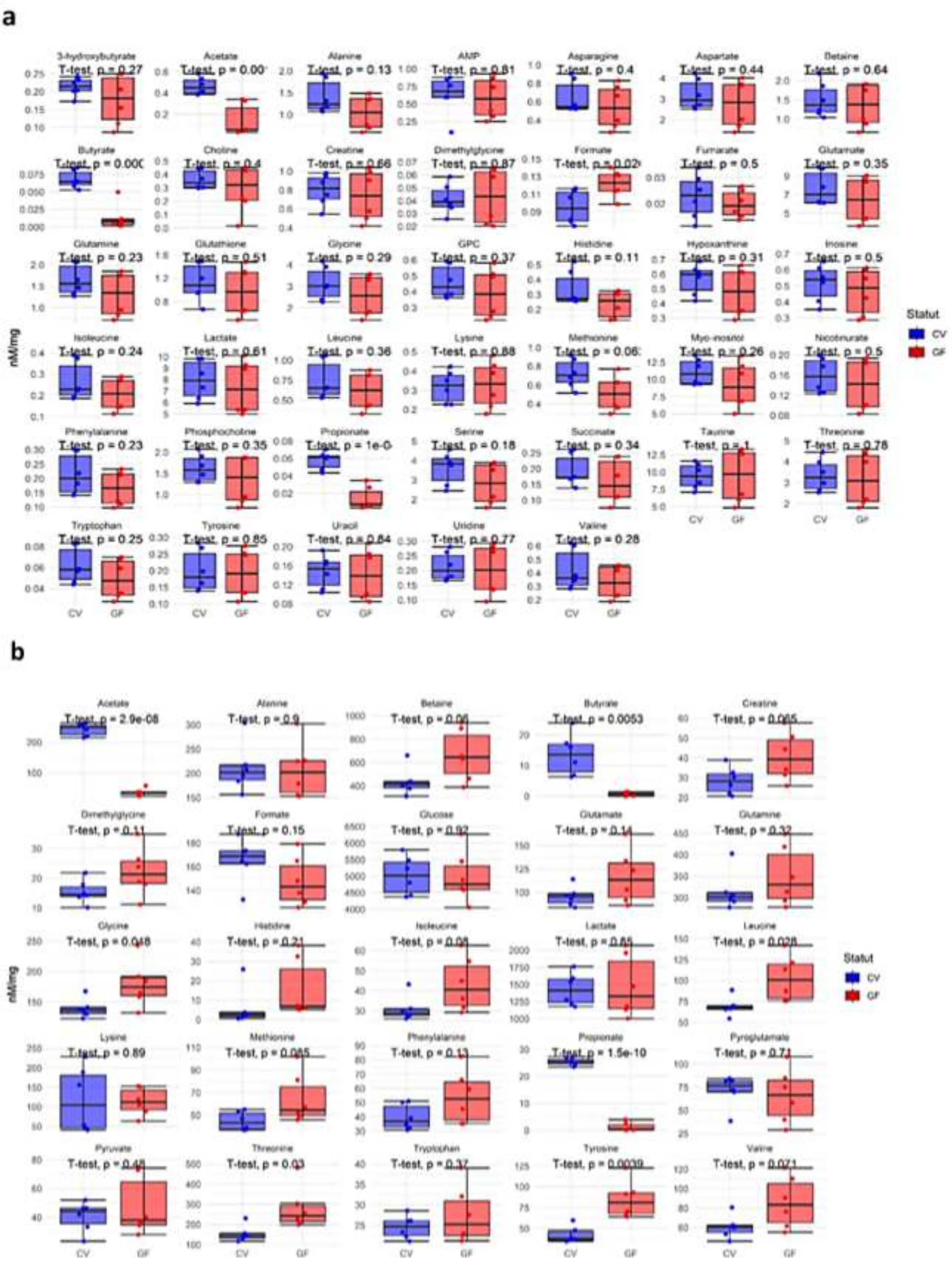

Supplementary Figure 5: Complete H^1-NMR dataset for the quantification of metabolites in chicken peripheral compartments. We used H1-NMR to analyse metabolites concentrations (nM/mg) in **spleen** (**a**) and **serum** (**b**) of 21-day-old conventional (CV) and germ-free (GF) chickens. Statistical comparisons of means for each metabolite were performed using an unpaired student's t-test, and p-values are indicated below the respective metabolite names. Each group comprised spleen or serum samples from 6 CV chickens and 6 GF chickens (n=6 biological replicates). The data shown are representative of one experiment out of two independent experiments. Data are represented as the mean ± SEM.

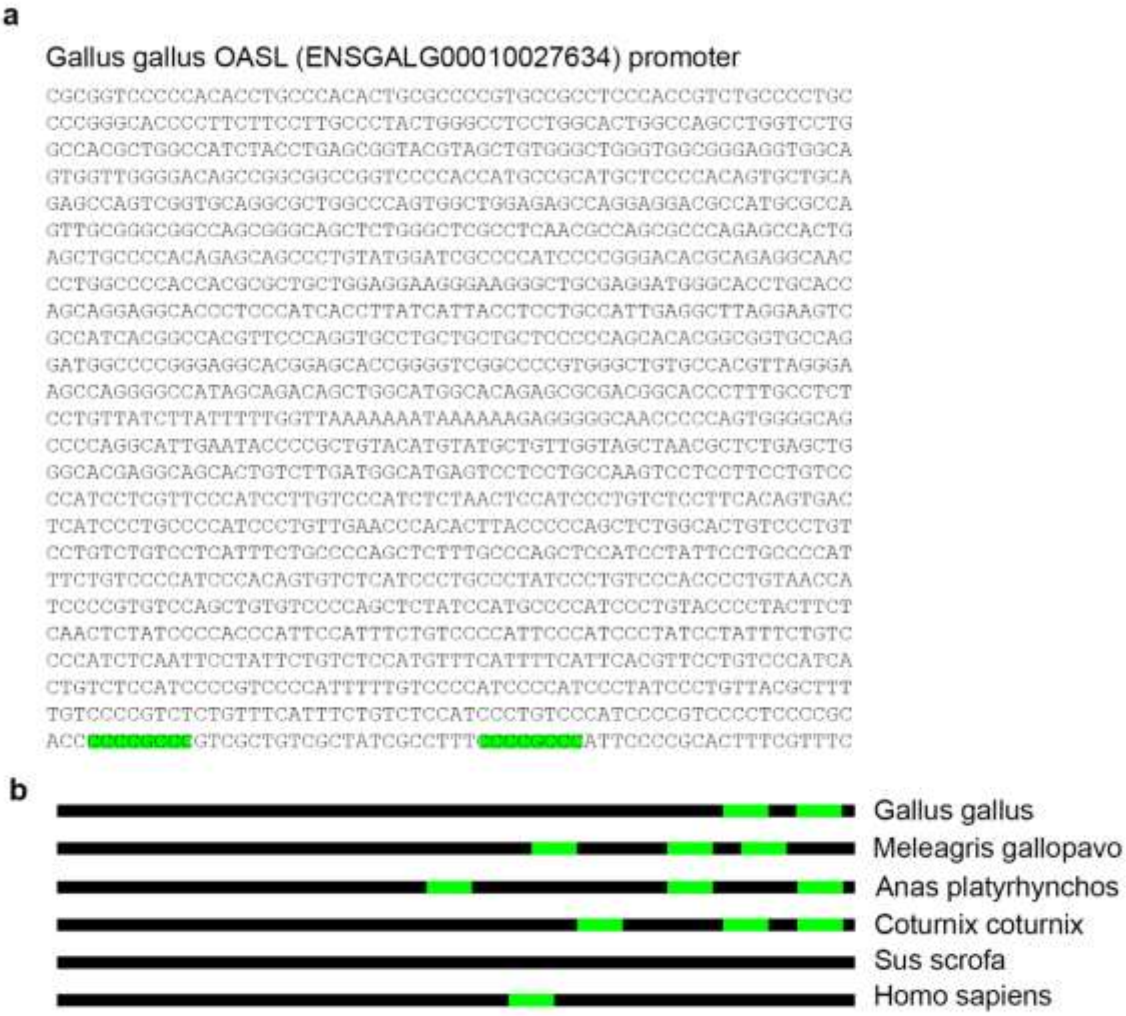

Supplementary Figure 6: Conservation of predicted SP1 binding sites in the promotors of avian OASL genes. a) The promoter of *Gallus gallus* OASL gene ENSGALG00010027634 (taken as 1500 bp upstream of the transcription start site), with sites of canonical SP1 binding sequences highlighted in green. **b**) Schematic diagram indicating positions of predicted SP1 binding sites (green) in the promoters of *OASL* genes from indicated species. Promoter regions were extracted from the following ensemble genes: *Gallus gallus* ENSGALG00010027634, *Melagris gallopavo* ENSMGAG00000018097, *Anas platyrynchos* ENSAPLG00030020164, *Coturnix coturnix* ENSCJPG00005012237; *Sus scrofa* ENSSSCG00000009921, *Homo sapiens* ENSG00000135114.

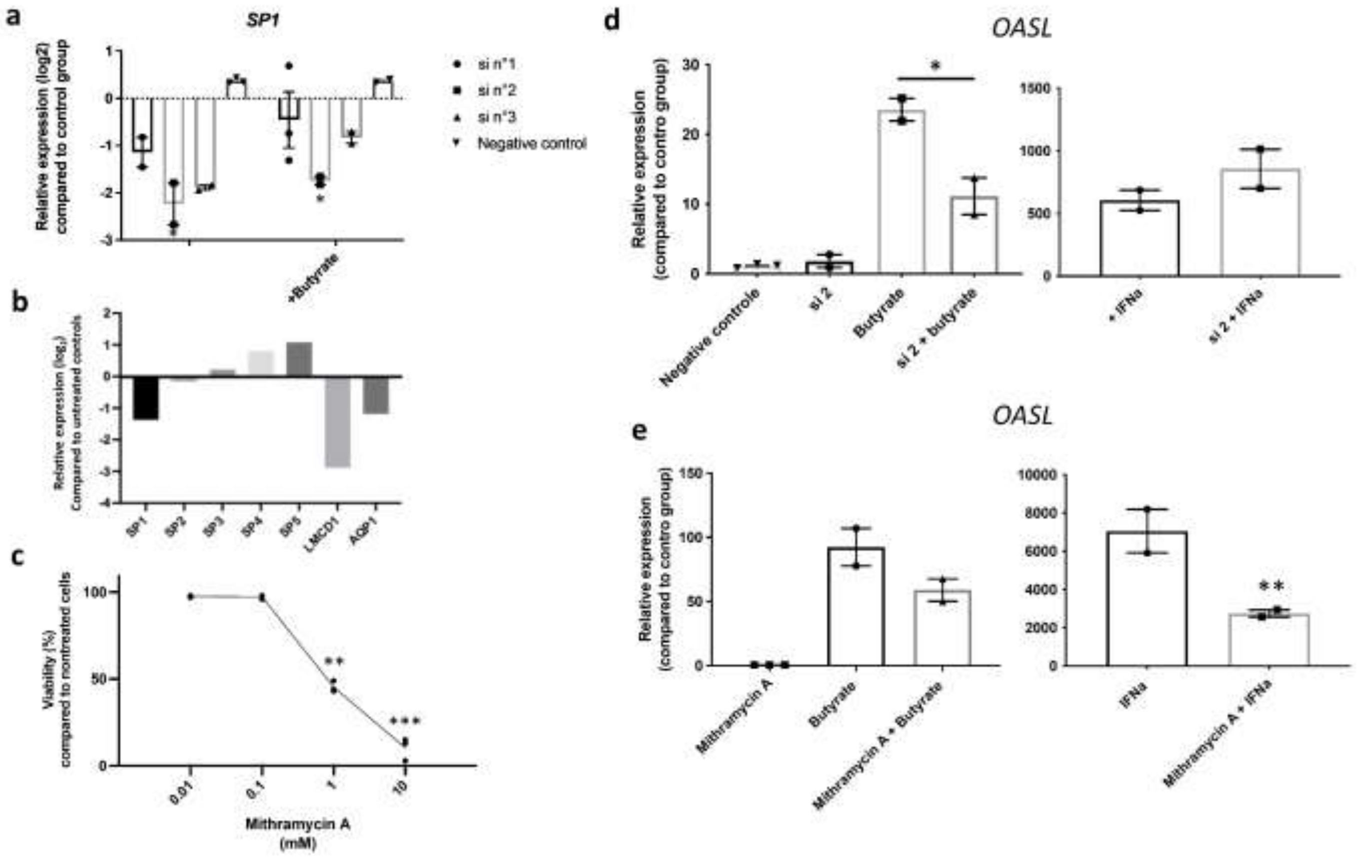

Supplementary Figure 7: *SP1* expression knockdown leads to a significantly diminished induction of *OASL* expression by butyrate in the CLEC213 cell line. To pinpoint the optimal *SP1* sequence targets for knockdown, three distinct siRNAs were developed, with a mixture of non-specific small RNAs as a negative control. (**a**) After 16 hours of transfection, siRNA n°2 exhibited the most pronounced reduction in *SP1* expression as evaluated by qPCR, validated by consistent decrease even with 3 mM butyrate treatment. (**b**) Specific *SP1* silencing with siRNA was confirmed by RNAseq analysis, leading to downregulation of Sp1-dependent genes *LMCD1* and *AQP1* (Padj < 0.05). (**c**) Mithramycin A induces cytotoxicity beyond 0.1 mM in the CLEC213 cell line. (**d**) *SP1* knockdown using siRNA reduces *OASL* expression induced by 3 mM butyrate for 16h, with no effect on IFNα-triggered *OASL* induction (recombinant chIFN-alpha, 50 ng/ml). (**e**) Pre-treatment with Mithramycin A at 0.1 mM reduces *OASL* expression induction by both butyrate (3mM) and IFN-α (50 ng/ml). One-way ANOVA followed by Tukey multiple comparison test was employed for statistical analyses, where *p<0.05, **p<0.01 and *** p<0.001. In panels **a**-**e**, an n=3 biological replicates is shown, in which each biological replicate is the mean of 3 technical replicates. Data in panel **b** are represented as the median. Data in panels **a**, **c**-**e** are represented as the mean ± SEM.

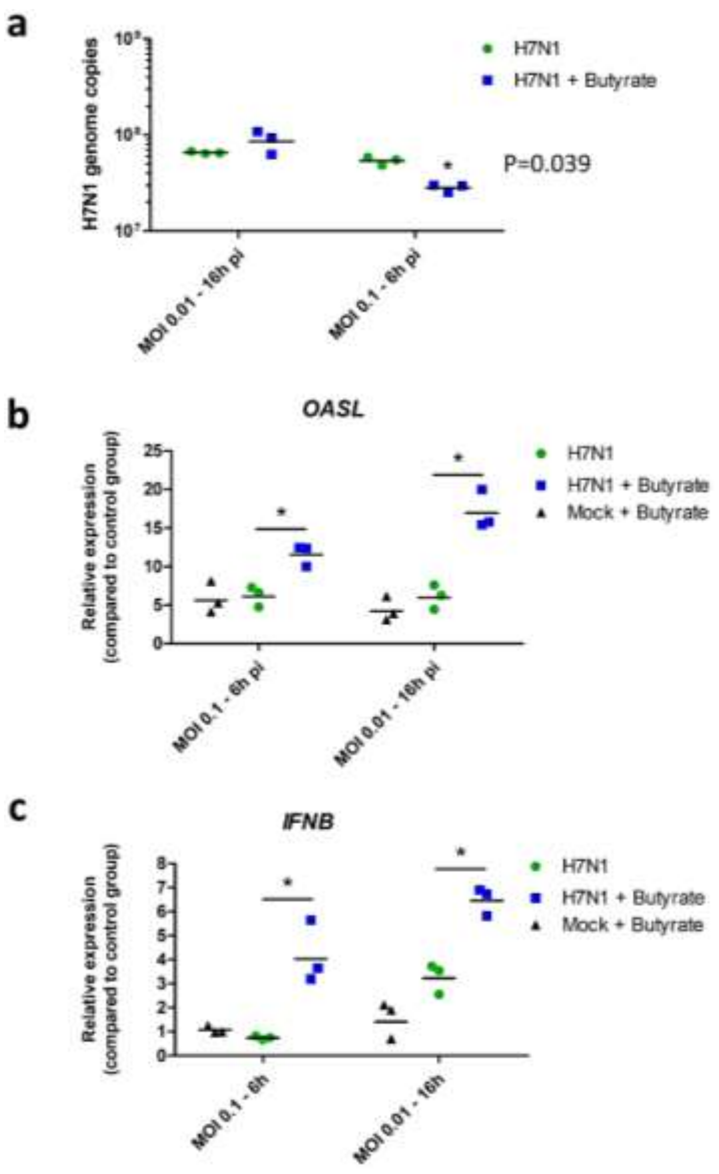

Supplementary Figure 8: Butyrate limits viral replication in chicken lung epithelial cells upon infection with an H7N1 avian influenza virus strain *via* an increased type-I IFN/ISG response. Treatment with butyrate (3 mM) for 16h reduces viral replication in CLEC213 cells infected with an H7N1 avian influenza virus strain. (**a**) Incubation with butyrate prior to infection (MOI 0.1 for 6 hours or MOI 0.01 for 16 hours) significantly reduces H7N1 genome copies compared to untreated infected cells. Conventional qPCR analysis revealed increased expression of *OASL* (**b**) and *IFNB* (**c**) in butyrate-treated cells infected with H7N1 at different MOIs and time-points. Statistical analyses were conducted using unpaired Student's t-test (**a**) and one-way ANOVA followed by Tukey multiple comparison test (**b**, **c**), where * indicates $p < 0.05$. In panels **a**-**c**, an n=3 biological replicates is shown, in which each biological replicate is the mean of 3 technical replicates. Data are shown as the median.

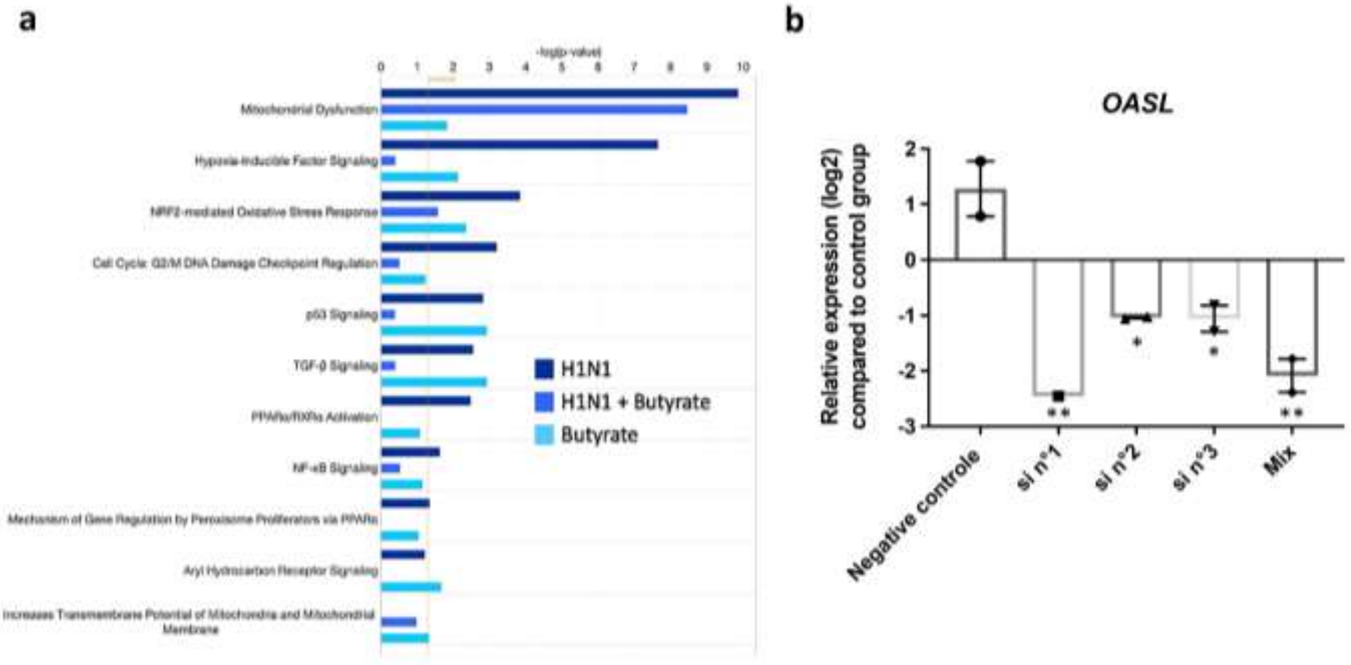

Supplementary Figure 9: Anti-viral effects of butyrate in chicken respiratory epithelial cells also rely on a wide transcriptional regulation. CLEC213 cells were treated with butyrate (3 mM) for 16h then infected with LPAIV H1N1 at an MOI 0.1 for 6h. (**a**) The comparison of canonical pathways affected by avian influenza H1N1 infection in CLEC213 cells, with or without pre-treatment with butyrate. RNAseq followed by ontology enrichment analysis using Ingenuity Analysis Pathway software revealed a reduced impact of the infection on various canonical pathways when cells were previously exposed to butyrate (3 mM). Butyrate mitigates H1N1-induced mitochondrial dysfunction, oxidative stress, and significantly dampens the activation of Hypoxia Inducible Factors (HIFs) and other pathways such as TGF-β, p53, and NF-kB. Fisher exact test was used for p-value calculation. (**b**) Transfection of CLEC213 cells with siRNAs targeting *OASL* transcripts reduces its expression during butyrate treatment (3 mM for 16h). siRNA n°1 shows the most significant effects, while a mix of all three siRNAs yields similar outcomes. One-way ANOVA followed by Tukey multiple comparison test was employed for statistical analysis (*p<0.05, **p<0.01). In panels **a** and **b**, an n=3 biological replicates was used, in which each biological replicate is the mean of 3 technical replicates. Data are shown as the mean ± SEM.

References

1. Pang, Z. *et al.* MetaboAnalyst 5.0: narrowing the gap between raw spectra and functional insights. *Nucleic Acids Research* **49**, W388–W396 (2021).
2. Hulsegge, I., Kommadath, A. & Smits, M. A. Globaltest and GOEAST: two different approaches for Gene Ontology analysis. *BMC Proc* **3**, S10 (2009).
