# Supplementary material for: The gut microbiota and its metabolite butyrate shape metabolism and antiviral immunity along the gut-lung axis in the chicken
Source: Commun Biol. 2024 Sep 20;7:1185. doi: 10.1038/s42003-024-06815-0 (PMC11413219; doi:10.1038/s42003-024-06815-0)
Supplement: Supplementary file 2 — Description of additional supplementary files [file 42003_2024_6815_MOESM2_ESM.pdf]

Description of Additional Supplementary Files

File name: Supplementary Data 1

Description: DEG from RNAseq in CV and GF chickens at 21 days of age.

File Name: Supplementary Data 2

Description: DEG from RNAseq in CLEC213 chicken lung epithelial cells.

File name: Supplementary Data 3

Description: Selected DEG from tissue RNAseq used for IPA analysis.
